# Cell-specific ablation of Hsp47 defines the collagen-producing cells in the injured heart

**DOI:** 10.1172/jci.insight.128722

**Published:** 2019-08-08

**Authors:** Hadi Khalil, Onur Kanisicak, Ronald J. Vagnozzi, Anne Katrine Johansen, Bryan D. Maliken, Vikram Prasad, Justin G. Boyer, Matthew J. Brody, Tobias Schips, Katja K. Kilian, Robert N. Correll, Kunito Kawasaki, Kazuhiro Nagata, Jeffery D. Molkentin

**Affiliations:** 1 Department of Pediatrics, Cincinnati Children’s Hospital Medical Center, and; 2 Department of Pathology, University of Cincinnati, Cincinnati, Ohio, USA.; 3 Department of Biological Sciences, University of Alabama, Tuscaloosa, Alabama, USA.; 4 Institute for Protein Dynamics, Kyoto Sangyo University, Kyoto, Japan.; 5 Howard Hughes Medical Institute, Cincinnati Children’s Hospital Medical Center, Cincinnati, Ohio, USA.

**Keywords:** Cardiology, Cardiovascular disease, Collagens, Fibrosis

## Abstract

Collagen production in the adult heart is thought to be regulated by the fibroblast, although cardiomyocytes and endothelial cells also express multiple collagen mRNAs. Molecular chaperones are required for procollagen biosynthesis, including heat shock protein 47 (Hsp47). To determine the cell types critically involved in cardiac injury–induced fibrosis the *Hsp47* gene was deleted in cardiomyocytes, endothelial cells, or myofibroblasts. Deletion of *Hsp47* from cardiomyocytes during embryonic development or adult stages, or deletion from adult endothelial cells, did not affect cardiac fibrosis after pressure overload injury. However, myofibroblast-specific ablation of *Hsp47* blocked fibrosis and deposition of collagens type I, III, and V following pressure overload as well as significantly reduced cardiac hypertrophy. Fibroblast-specific *Hsp47*-deleted mice showed lethality after myocardial infarction injury, with ineffective scar formation and ventricular wall rupture. Similarly, only myofibroblast-specific deletion of *Hsp47* reduced fibrosis and disease in skeletal muscle in a mouse model of muscular dystrophy. Mechanistically, deletion of *Hsp47* from myofibroblasts reduced mRNA expression of fibrillar collagens and attenuated their proliferation in the heart without affecting paracrine secretory activity of these cells. The results show that myofibroblasts are the primary mediators of tissue fibrosis and scar formation in the injured adult heart, which unexpectedly affects cardiomyocyte hypertrophy.

## Abstract 2

Deletion of the Hsp47 gene in cardiac myofibroblasts, but not myocytes or endothelial cells, selectively inhibits fibrosis in mice following injury.

## Introduction

The extracellular matrix (ECM) in the heart is comprised of diverse structural proteins that provide a rigid but dynamic framework that mechanically supports contracting cardiomyocytes. The ECM in the adult heart is composed predominantly of collagen type I, although other fibrillar and nonfibrillar collagens are present (1). Other critical components of the ECM in the adult heart include fibronectin, laminin, elastins, diverse proteoglycans, hyaluronan, and matricellular proteins, such as tenascin-C, SPARC, periostin, osteopontin, and CCN proteins (2, 3). The fibrillar collagens typically serve as the primary support network in the adult heart, and they also likely contribute to mechanosensation as part of cardiomyocyte and fibroblast reactive signaling (4). After myocardial infarction (MI), fibrillar collagen deposition is dramatically upregulated, allowing for rapid scar formation to preserve the structural integrity of the injured ventricular chamber (5). In addition, the cardiac hypertrophic response can involve induction of collagens type I and III along with a marked reduction of collagen degradation (6–8). While initially an adaptive response, excessive myocardial collagen deposition after pressure overload–induced hypertrophy or from a previous ischemic injury event predisposes to heart failure with diminished ventricular compliance, reduced diffusion efficiency within the tissue, and maladaptive structural remodeling (8–12).

Several molecular chaperones are involved in procollagen biosynthesis and folding within the endoplasmic reticulum (ER). These include BiP/Grp78 (13), Grp94 (14), protein disulfide isomerase (PDI) (15) and heat shock protein 47 (Hsp47) (16). Hsp47 is a stress-inducible collagen-specific molecular chaperone involved in the processing and/or secretion of procollagen (17–19). Hsp47 is upregulated in various fibrotic diseases of the lung (20), liver (21), kidney (22), and heart (23). Moreover, increased expression of human Hsp47 was reported in fibrotic lesions of idiopathic pulmonary fibrosis (24), fibrotic transplanted kidney (25), and peritoneal sclerosis (26). Global *Hsp47* gene–deleted mice are embryonic lethal and the embryos show deficient maturation of collagen type I and IV (27). Chondrocyte-specific disruption of *Hsp47* causes defective endochondral bone formation and a marked reduction in collagen type II and type XI within the cartilage (28).

While fibroblasts are assumed to be the primary source of ECM deposition and tissue fibrosis (29, 30), other cell types are known to generate ECM components and collagen. For example, endothelial cells produce ECM proteins, including collagens (31–33), while collagen type I, III, and VI synthesis was identified in cardiomyocytes (34, 35), with increased collagen1a1 mRNA during heart injury (36). Here, we utilized *Hsp47-loxP*–targeted mice to inducibly disrupt this gene in adults to identify the critical cell types responsible for disease-based fibrosis and acute scar formation. Our study identifies the myofibroblast as the primary mediator of collagen generation and the fibrotic response in the adult heart after injury. Furthermore, inhibition of myofibroblast collagen production secondarily effected the ability of the mouse heart to effectively hypertrophy following pressure overload stimulation.

## Results

### Hsp47 deletion attenuates collagen type I secretion in cultured fibroblasts.

**Figure 1:**
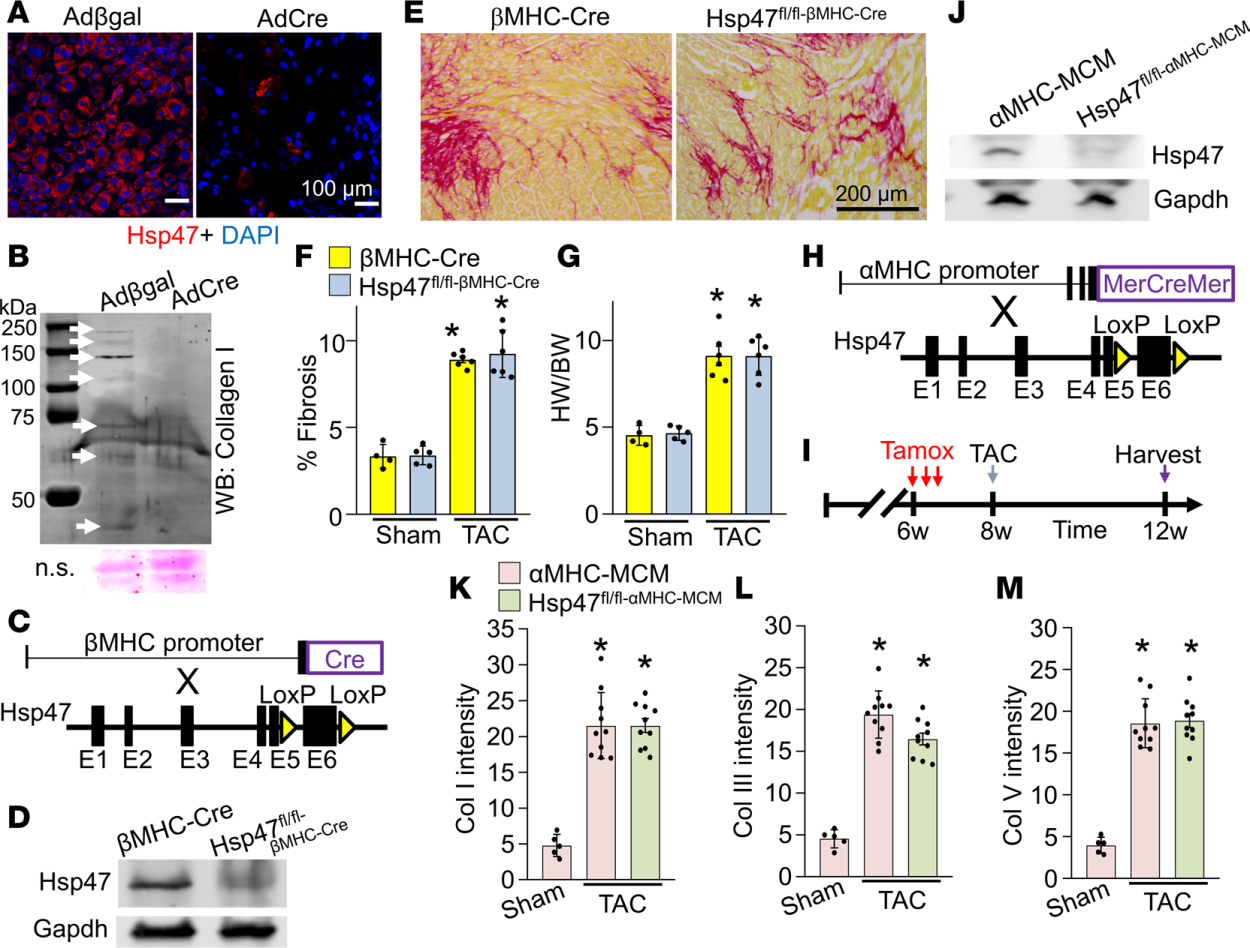
Cardiomyocyte-specific deletion of *Hsp47* in the mouse heart does not block maladaptive fibrosis with TAC. (**A**) Representative immunostaining for Hsp47 (red fluorescence) in cultured cardiac fibroblasts 72 hours after infection with Adβgal and AdCre. Nuclei are stained blue with DAPI. Scale bar: 100 μm. (**B**) Western blot showing levels of secreted collagen type I in the culture media of heart fibroblasts 72 hours after infection with either Adβgal or AdCre. White arrows show collagen isoforms. Molecular weight migration standard and sizes are also shown. Nonspecific (n.s.) Ponceau staining (pink) is shown as a processing and loading control. (**C**) Schematic representation of breeding βMHC-Cre–transgenic mice with *Hsp47-loxP–*targeted mice. (**D**) Western blot analysis for Hsp47 isolated from fractionated cardiomyocytes of the 2 genotypes of mice shown. Gapdh is shown as the loading control. (**E** and **F**) Picrosirius red–stained histological heart sections, and quantitation of the area of fibrosis (red) in hearts from the indicated genotypes of mice with the βMHC-Cre transgene after 4 weeks of TAC injury. Average fibrotic area ± SEM, *n* = 5–8 mice in each group, *P < 0.05 versus sham-operated βMHC-Cre mice. *P* values were calculated by 1-way ANOVA with Tukey’s post hoc test. Scale bar: 200 μm (**G**) Heart-weight-to-body-weight (HW/BW) ratio in mice after 4 weeks of TAC. *n* = 5–8 in each group. *P < 0.05 versus βMHC-Cre sham mice. (**H**) Breeding scheme of αMHC-MCM–transgenic mice with *Hsp47-loxP*–targeted mice. (**I**) Experimental regimen whereby mice were subjected to TAC injury or sham procedure for 4 weeks along with tamoxifen treatment by injection (vertical red arrows). (**J**) Western blot analysis for Hsp47 isolated from adult heart fractionated cardiomyocytes of the 2 genotypes shown. Gapdh is shown as a loading control. (**K**, **L**, and **M**) Quantitation of collagens type I, III, and V, respectively, from immunohistochemical heart images from WT αMHC-MCM mice versus *Hsp47* cardiomyocyte-specific mice, as shown in Supplemental Figure 3. Ten random histological sections from each mouse heart were imaged and quantified from 5–10 mice each per group. *P < 0.05 versus αMHC-MCM Sham. *P* values were calculated with 1-way ANOVA with Tukey’s post hoc test.

To examine the role of Hsp47 in collagen secretion we first generated primary heart fibroblasts from mice with homozygous loxP-site (fl) targeted alleles for *Hsp47*. These primary heart fibroblasts were infected with a Cre-expressing adenovirus (AdCre) or with a β-galactosidase–expressing (Adβgal-expressing) control adenovirus (Figure 1A). Immunocytochemistry 72 hours after recombinant adenoviral infection showed nearly a complete deletion of Hsp47 protein from *Hsp47^fl/fl^* double homozygous cardiac fibroblasts with AdCre (Figure 1A). Next, Adβgal- and AdCre-infected *Hsp47^fl/fl^* cultured cardiac fibroblasts were stimulated for 18 hours with a combination of ascorbic acid and TGF-β to induce collagen generation. The media were collected, concentrated, and assayed for collagen type I isoforms by Western blotting, which showed a near-complete absence with AdCre (Figure 1B). Consistent with these findings, reduced secretion of collagen in *Hsp47*-deleted fibroblasts was accompanied by an increase in unfolded collagen content, as detected with a collagen-hybridizing peptide (CHP) that detects the unfolded collagen triple helix (37). Immunohistological analysis of CHP showed increased accumulation of unfolded collagen content in *Hsp47*-deleted fibroblasts, as compared with control fibroblasts, 72 hours after *Hsp47* deletion (Supplemental Figure 1A; supplemental material available online with this article; https://doi.org/10.1172/jci.insight.128722DS1). Given these results, we were concerned that deletion of *Hsp47* might compromise the ER and general ability of fibroblasts to secrete proteins. Direct assessment of a broad array of secreted factors in cultured cardiac fibroblasts with an immune-based profiling assay showed no general defects (Supplemental Figure 2A). More specifically, deletion of *Hsp47* with AdCre only affected the secretion of a few factors/cytokines of 111 examined (Supplemental Figure 2, B and C). For example, loss of *Hsp47* enhanced the secretion of 11 factors/cytokines, including matrix metalloproteinase 3 (MMP3), osteoprotegerin, and pentraxin 2 (Supplemental Figure 2, D–F), while only the secretion of IL-17 was significantly reduced (Supplemental Figure 2G). Overall, these results suggest that Hsp47 specifically regulates the secretion of ECM-associated collagens in cultured cardiac fibroblasts and its deletion does not otherwise compromise the secretory function of these fibroblasts.

### Cardiomyocyte-specific deletion of Hsp47 does not modulate collagen generation after injury.

Global disruption of the *Hsp47* gene in the mouse caused early developmental lethality just prior to embryonic day 12 (27). To investigate whether cardiomyocyte-mediated collagen deposition was involved in heart development and function, we crossed β-myosin heavy chain (βMHC) Cre-containing transgenic mice (38) with *Hsp47^fl/fl^* targeted mice (Figure 1C). Hsp47 protein was identified in the cardiomyocyte fraction from hearts of βMHC-Cre control mice by Western blotting, while protein levels were reduced by more than 80% from hearts of *Hsp47*^fl/fl-*β*MHC-Cre^** mice (Figure 1D). Hearts from *Hsp47*^fl/fl-*β*MHC-Cre^** mice appeared otherwise without disease based on measurements employed here, suggesting that cardiomyocytes are not likely a significant source of collagen production during development. These same mice as young adults were also subjected to 4 weeks of pressure overload stimulation by transverse aortic constriction (TAC) surgery, which showed that controls (βMHC-Cre) and *Hsp47*^fl/fl-βMHC-Cre^ mice displayed comparable levels of induced myocardial fibrosis (Figure 1, E and F) as well as similar cardiac function and equal induction of the hypertrophic response (Figure 1G and Supplemental Table 3).

*Hsp47* was also deleted from the adult heart using the tamoxifen-inducible α-myosin heavy chain (αMHC) MerCreMer-containing (MCM-containing) transgene (ref. 39 and Figure 1H). Tamoxifen was injected for 3 consecutive days 2 weeks before the TAC surgery procedure (Figure 1I). Western blotting with lysates from isolated adult cardiomyocytes from hearts of *Hsp47*^fl/fl-*α*MHC-MCM^** mice showed a greater than 90% loss of Hsp47 protein compared with control αMHC-MCM mice (Figure 1J). Similar to embryonic deletion of *Hsp47* from cardiomyocytes, deletion of *Hsp47* specifically from adult cardiomyocytes of mice showed no ability to reduce or otherwise alter the pressure overload–induced fibrotic response compared with αMHC-MCM controls (Figure 1, K–M, and Supplemental Figure 3). Similarly, hydroxyproline assay–based quantitation of fibrosis showed no reduction after TAC with adult cardiomyocyte-specific deletion of *Hsp47* (Supplemental Figure 4A). Histological quantification using the CHP (37) assay also showed similar accumulation of unfolded collagen content in cardiomyocyte-specific *Hsp47*-deleted and control hearts (Supplemental Figure 4B). In conclusion, cardiomyocyte-specific inhibition of collagen secretion had no effect on the ability of the adult heart to mount a fulminant fibrotic response following pressure overload.

### Endothelial-specific deletion of Hsp47 mildly reduces collagen type I without affecting total heart fibrosis.

**Figure 2:**
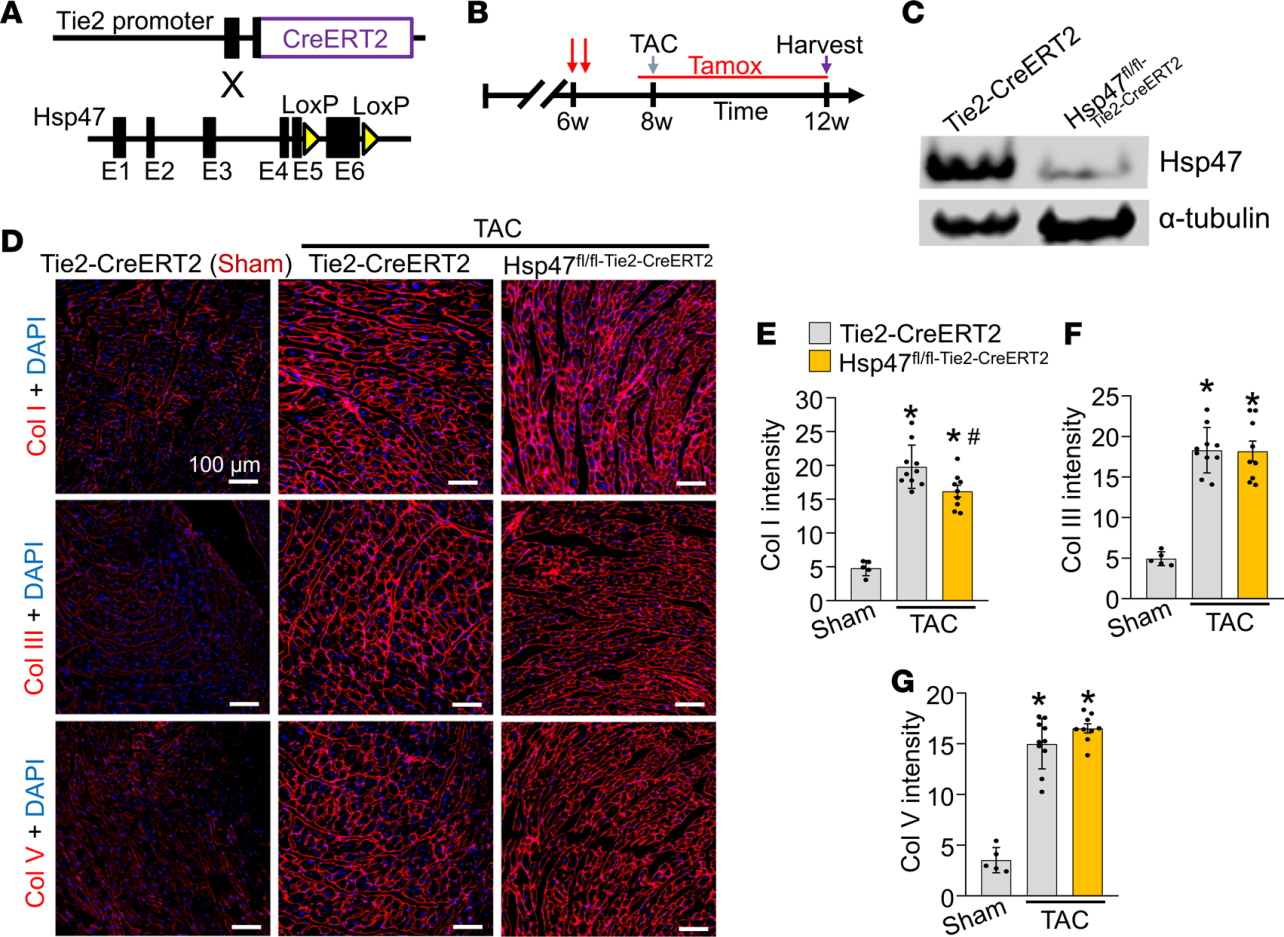
Endothelial-specific deletion of *Hsp47* in the heart. (**A**) Schematic of breeding tamoxifen-inducible Tie2-CreERT2–transgenic mice with *Hsp47-loxP*–targeted mice. (**B**) Experimental regimen for mice subjected to TAC or a sham procedure for 4 weeks. Mice were injected at 6 weeks of age 2 times with tamoxifen and then put on tamoxifen chow before 8 weeks of age through harvesting at 12 weeks. (**C**) Western blot analysis for Hsp47 from endothelial cells isolated by FACS from the genotypes shown. Gapdh is a loading control. (**D**–**G**) Immunohistochemical heart images stained (scale bar: 100 μm) and quantified for collagen type I, III, and V from Tie2-CreERT2–transgenic mice and *Hsp47* endothelial-specific deleted mice. Mice were subjected to TAC as shown in **B**. Quantitation shows mean intensity of immunoreactivity taken from 10 random histological sections from 5–10 mice in each group. *P < 0.05 versus Tie2-CreERT2 Sham. ^#^*P* < 0.05 versus Tie2-CreERT2 TAC. *P* values were calculated with 1-way ANOVA with Tukey’s post hoc test.

Endothelial cells were reported to produce ECM proteins, including collagens; however, the contribution of these cells to myocardial fibrosis and progression of heart disease has not been evaluated in vivo (31–33). To investigate the endothelial cell contribution to cardiac fibrosis *Hsp47^fl/fl^* mice were crossed with the endothelial cell–specific and inducible transgenic line, Tie2-CreERT2 (ref. 40 and Figure 2A). Note that this endothelial-specific Cre mouse line uses the regulatory sequences of the *Tek* gene, which was reported to show some degree of Cre recombinase activity in the hematopoietic lineage (41). We also crossed the Tie2-CreERT2 line with a single *Rosa26-loxP*-Stop-loxP-EGFP (*R26^eGFP^*) reporter allele to allow Cre-dependent EGFP expression in targeted endothelial cells as previously described (40). Mice were injected with tamoxifen at 6 weeks of age and then placed on tamoxifen-laden food 48 hours prior to surgery and continued thereafter so that deletion of *Hsp47* would be ongoing as new endothelial cells arose during the pressure overload period (Figure 2B). Western blotting showed that EGFP-purified endothelial cells from the heart indeed expressed abundant Hsp47 protein, which was effectively deleted with the Tie2-CreERT2 transgene 4 weeks after TAC surgery (Figure 2C). Immunohistological analysis of heart sections showed a slight but significant reduction in collagen type I staining intensity in hearts of endothelial-specific *Hsp47* gene–deleted mice compared with controls (Figure 2, D and E), although collagen type III and V were not reduced (Figure 2, D, F, and G). Additionally, Picrosirius red staining (data not shown) and hydroxyproline quantification showed a similar fibrotic response in both endothelial-specific *Hsp47*-deleted and control hearts with 4 weeks of pressure overload (Supplemental Figure 4A). Finally, the content of unfolded collagens evaluated by CHP assay showed no difference between endothelial-specific *Hsp47*-deleted and control hearts (Supplemental Figure 4B). Thus, endothelial cells are not a primary cell type that mediates significant cardiac fibrosis with pressure overload.

### Myofibroblast-specific Hsp47-deleted mice show reduced collagen deposition with TAC.

**Figure 3:**
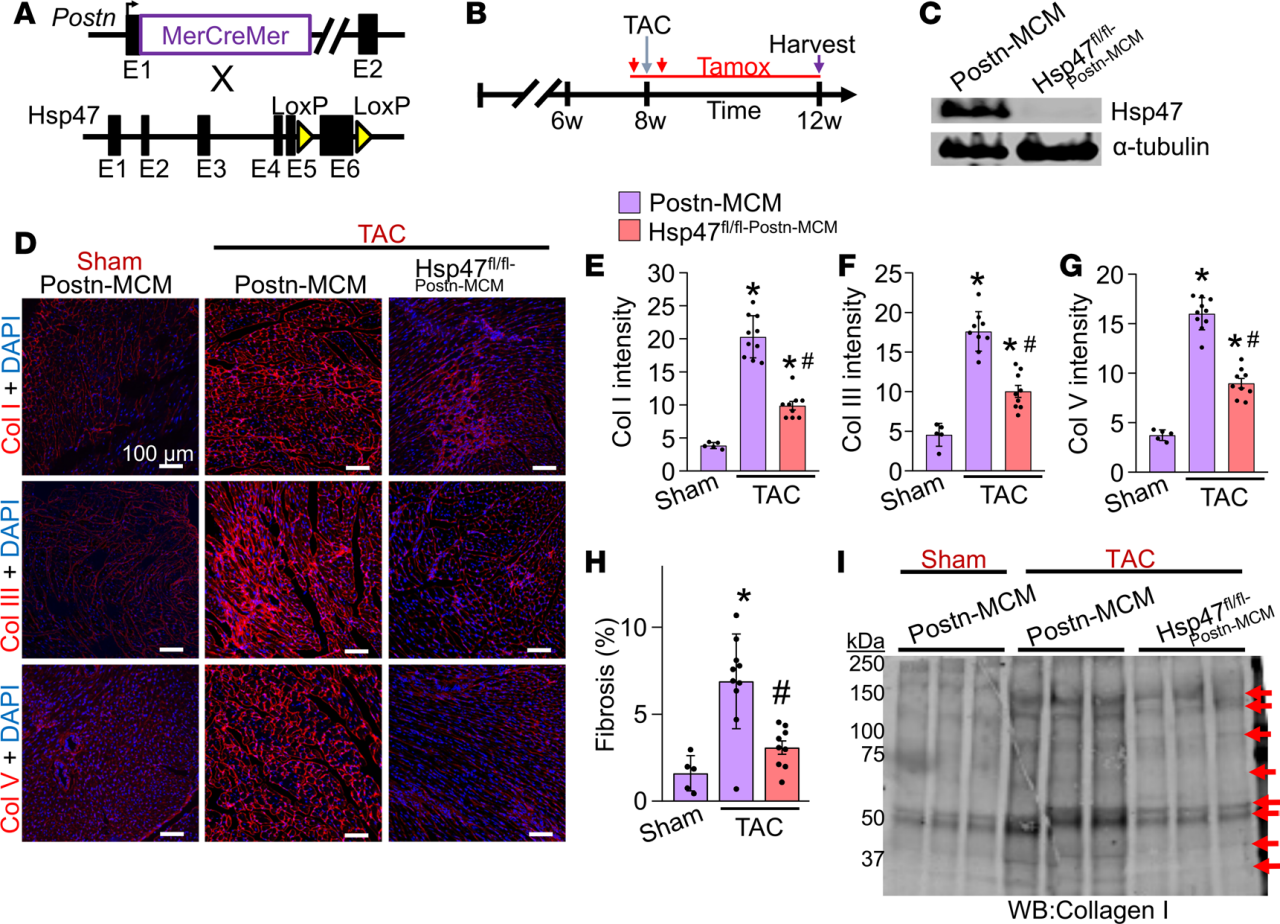
Myofibroblast-specific deletion of *Hsp47* in the heart reduces myocardial fibrosis after TAC. (**A**) Schematic representation of *Postn-MerCreMer*–targeted (MCM-targeted) mice crossed with *Hsp47-loxP*–targeted mice. (**B**) Experimental regimen of tamoxifen injections (red vertical arrows) and feed treatment (red horizontal line) in mice subjected to TAC for 4 weeks. (**C**) Western blot analysis for Hsp47 and α-tubulin from 500,000 EGFP^+^ cells isolated by FACS from hearts of the 2 genotypes of mice shown (*R26^eGFP^* reporter was also present). (**D**–**G**) Representative immunohistochemistry (scale bar: 100 μm) of heart tissue sections and quantitation for collagen type I, III, and V from hearts of *Postn*-MCM control mice and *Hsp47* myofibroblast-specific deleted mice using the *Postn*-*MCM* allele after 4 weeks of TAC. (**H**) Quantitation from Picrosirius red–stained histological sections in hearts from the indicated genotypes of mice after 4 weeks of TAC injury. *P < 0.05 versus *Postn*-MCM Sham. ^#^*P* < 0.05 versus *Postn*-*MCM* TAC. *n* = 5–10 mice in each group. *P* values were calculated using a 1-way ANOVA with Tukey’s post hoc test. (**I**) Western blot analysis for collagen type I from heats of sham and TAC-operated mice using cardiac extracellular matrix–specific protein preparations from the indicated genotypes of mice. The red arrows show collagen isoforms. Position of molecular weight standards (kDa) are shown on the left.

Here we used *Postn* gene–targeted mice containing the same tamoxifen-inducible MCM cDNA (36) and crossed them with the *Hsp47^fl/fl^* targeted mice to delete *Hsp47* in only myofibroblasts (activated fibroblasts) after cardiac injury (Figure 3A). The *R26^eGFP^* reporter allele was also included to allow tracking and isolation of all myofibroblasts from the heart (36). Mice were subjected to pressure overload for 4 weeks by TAC in the presence of a tamoxifen diet so that the inducible MCM protein continuously produced recombination in existing and newly activated fibroblasts (Figure 3B). Western blotting of lysates from EGFP-expressing FACS-sorted myofibroblasts from *Hsp47^fl/fl-Postn-MCM/+^R26^eGFP/+^* mice showed efficient deletion of Hsp47 protein compared with control *Postn-MCM* myofibroblasts (Figure 3C). Levels of fibrillar collagens type I, III, and V were assessed by immunohistochemistry (Figure 3D), which showed essentially no induction after 4 weeks of TAC with myofibroblast-specific deletion of *Hsp47* compared with *Postn-MCM* controls (Figure 3, D–G). We also assessed TAC-induced fibrosis in Picrosirius red–stained cardiac histological sections (Figure 3H) and the content of collagen type I in whole heart ECM protein preps by Western blotting in *Hsp47^fl/fl-Postn-MCM/+^* mice, which again showed a significant reduction versus TAC-operated *Postn-MCM* control mice (Figure 3I). Biochemical hydroxyproline assays (Supplemental Figure 4A) and histological assays for CHP intensity also showed that myofibroblast-specific *Hsp47*-deleted hearts had significantly reduced levels compared with controls hearts after 4 weeks of pressure overload (Supplemental Figure 4B). Consistent with these results, although Hsp47 is expressed in both cardiomyocytes and fibroblasts, after mechanical stretching, Hsp47 expression was induced only in fibroblasts but not in cardiomyocytes (Supplemental Figure 5, A and B). Taken together, these results indicate that cardiac myofibroblasts, but not cardiomyocytes or endothelial cells, are the primary cell-type in the heart responsible for mediating the pathologic fibrotic response.

We also used *Tcf21* gene–targeted mice containing the same tamoxifen-inducible MCM cDNA, which were crossed with the *Hsp47^fl/fl^* targeted mice to delete Hsp47 in only fibroblasts at baseline before cardiac injury (Supplemental Figure 6A). Mice were fed a tamoxifen diet 1 week before surgery and then were subjected to pressure overload for 4 weeks by TAC (Supplemental Figure 6B). Levels of myocardial fibrosis were assessed by Picrosirius red staining (Supplemental Figure 6, C and D), which showed a significant reduction of fibrosis with fibroblast-specific deletion of *Hsp47* compared with *Tcf21-MCM* controls after 4 weeks of TAC injury. Taken together, these results confirm that deletion of Hsp47 in cardiac fibroblasts or myofibroblasts reduces myocardial fibrosis with pressure overload.

### Loss of Hsp47 from myofibroblasts in dystrophic skeletal muscle attenuates fibrosis and enhances muscle performance.

**Figure 4:**
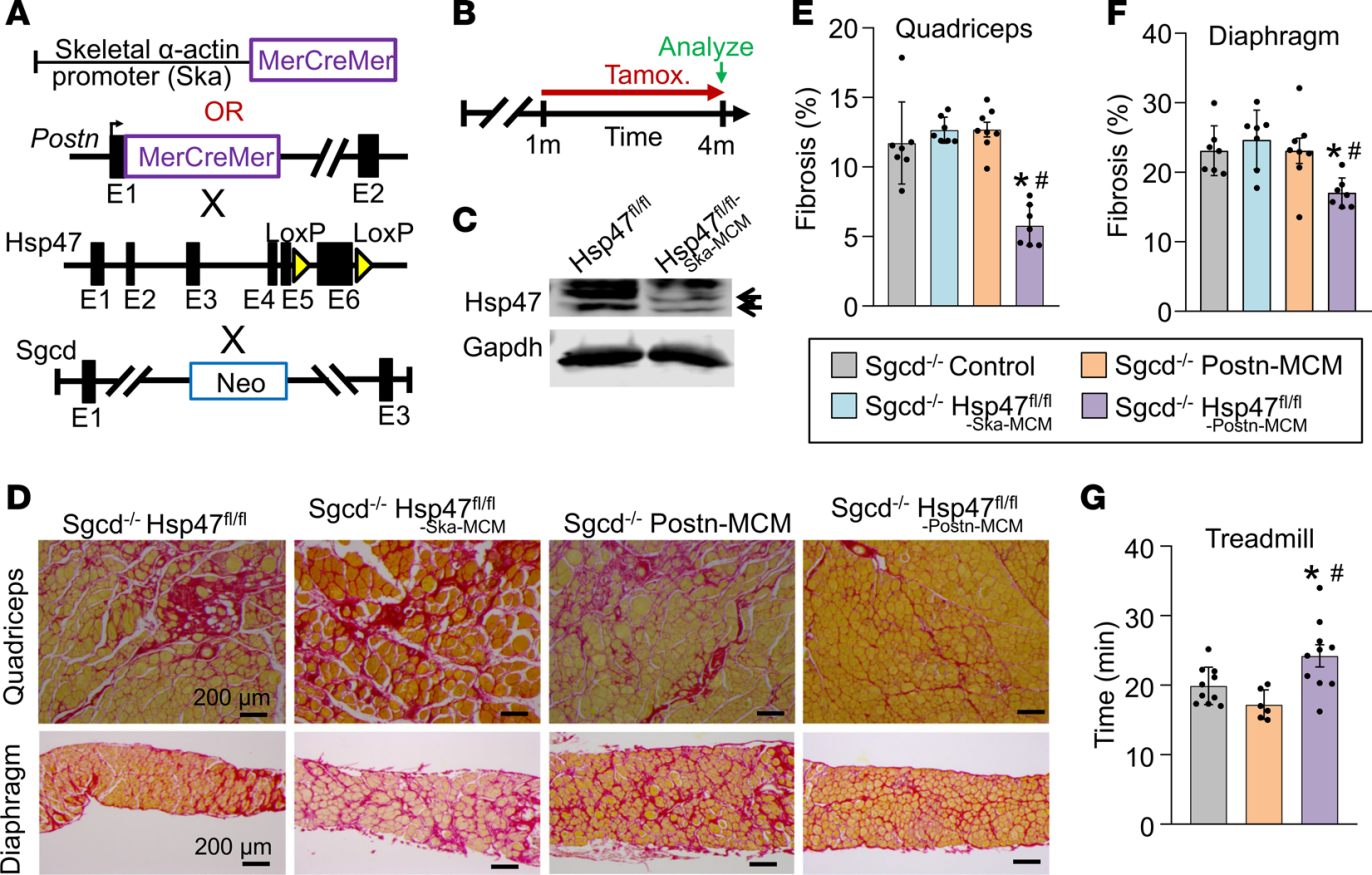
Myofibroblast-specific but not myofiber-specific deletion of *Hsp47* in skeletal muscle reduces muscular dystrophy–dependent tissue fibrosis. (**A**) Schematic of the MCM cDNA driven by the human skeletal α-actin promoter (myofiber-specific) or the myofibroblast-specific *Postn* genetic locus to delete the *Hsp47* gene with tamoxifen treatment. These lines were crossed into the δ-sarcoglycan–null (*Sgcd*^–/–^) background. (**B**) Experimental tamoxifen dosing regimen administered in the feed. (**C**) Western blot analysis for total Hsp47 protein using whole muscle protein lysates from the quadriceps of mice of the indicated genotypes. *n* = 6 mice per group. Gapdh is shown as a loading control. (**D**) Representative Picrosirius red–stained histological sections from quadriceps and diaphragm from 4-month-old mice of the indicated genotypes. Scale bar: 200 μm. (**E** and **F**) Quantitation of fibrosis from Picrosirius red–stained histological sections from quadriceps and diaphragm of 4-month-old mice of the indicated genotypes. *n* = 7 mice in each group. (**G**) Average time spent running on a treadmill of 4-month-old mice of the indicated genotypes. *n* = 6–10 in each group. Significance was determined using *P* values calculated by 1-way ANOVA with Tukey’s post hoc test. *P < 0.05 versus *Sgcd*^–/–^-control. ^#^*P* < 0.05 versus *Sgcd^–/–^ Hsp47^fl/fl-Ska-MCM^*. The legend applies to **E**, **F** and **G**.

Muscular dystrophy is a longstanding chronic disease characterized by accumulation of collagen and ECM (42), although the direct involvement of the tissue-resident fibroblast in skeletal muscle fibrosis is unclear. Here we used a mouse model of limb-girdle muscular dystrophy lacking the δ-sarcoglycan gene (*Sgcd^–/–^*), which shows continuous myofiber necrosis in skeletal muscle, inflammation, and fibrosis that accumulates over time (43). As in heart, skeletal muscle cells also express select collagen mRNAs (44, 45), so it is unclear how much of disease-based fibrosis is due to myofiber collagen production versus activated fibroblasts. Here we inducibly deleted the *Hsp47* gene in either myofibers or myofibroblasts of *Sgcd*-null mice. We have previously characterized a transgene containing the MCM cDNA driven by the skeletal muscle–specific skeletal α-actin promoter (Ska-MCM) (46). Tamoxifen was given at 1 month of age with tamoxifen-laden food until harvest at 4 months of age (Figure 4, A and B). We have previously shown that the *Postn-MCM* allele is efficiently expressed in activated fibroblasts in injured skeletal muscle (36). Western blot analysis performed on whole muscle protein lysates from *Sgcd^–/–^ Hsp47^fl/fl-Ska-MCM^* mice showed a greater than 80% loss of endogenous Hsp47 protein in myofibers compared with *Hsp47^fl/fl^* control mice (Figure 4C). Histological analysis of muscle disease showed that loss of *Hsp47* from the myofibers did not reduce dystrophy-dependent fibrosis compared with controls (Figure 4, D–F). In contrast, deletion of *Hsp47* from *Sgcd^–/–^* mice using the *Postn*-MCM allele showed a significant reduction in fibrosis of both the quadriceps and diaphragm (Figure 4, D–F). Mice were then challenged to run on a treadmill as an indirect measure of muscle performance. Consistent with less fibrosis, *Sgcd^–/–^* mice with *Hsp47* deleted in activated fibroblasts performed significantly better compared with the appropriate controls (Figure 4G). These results confirm and extend the results observed in the heart, whereby the activated, tissue-resident fibroblast is the most critical mediator of interstitial fibrosis in response to disease stimulation in striated muscle.

### Reduced fibrosis in myofibroblast-specific Hsp47-deleted mice attenuates myocardial hypertrophy and increases cardiac rupture after MI injury.

**Figure 5:**
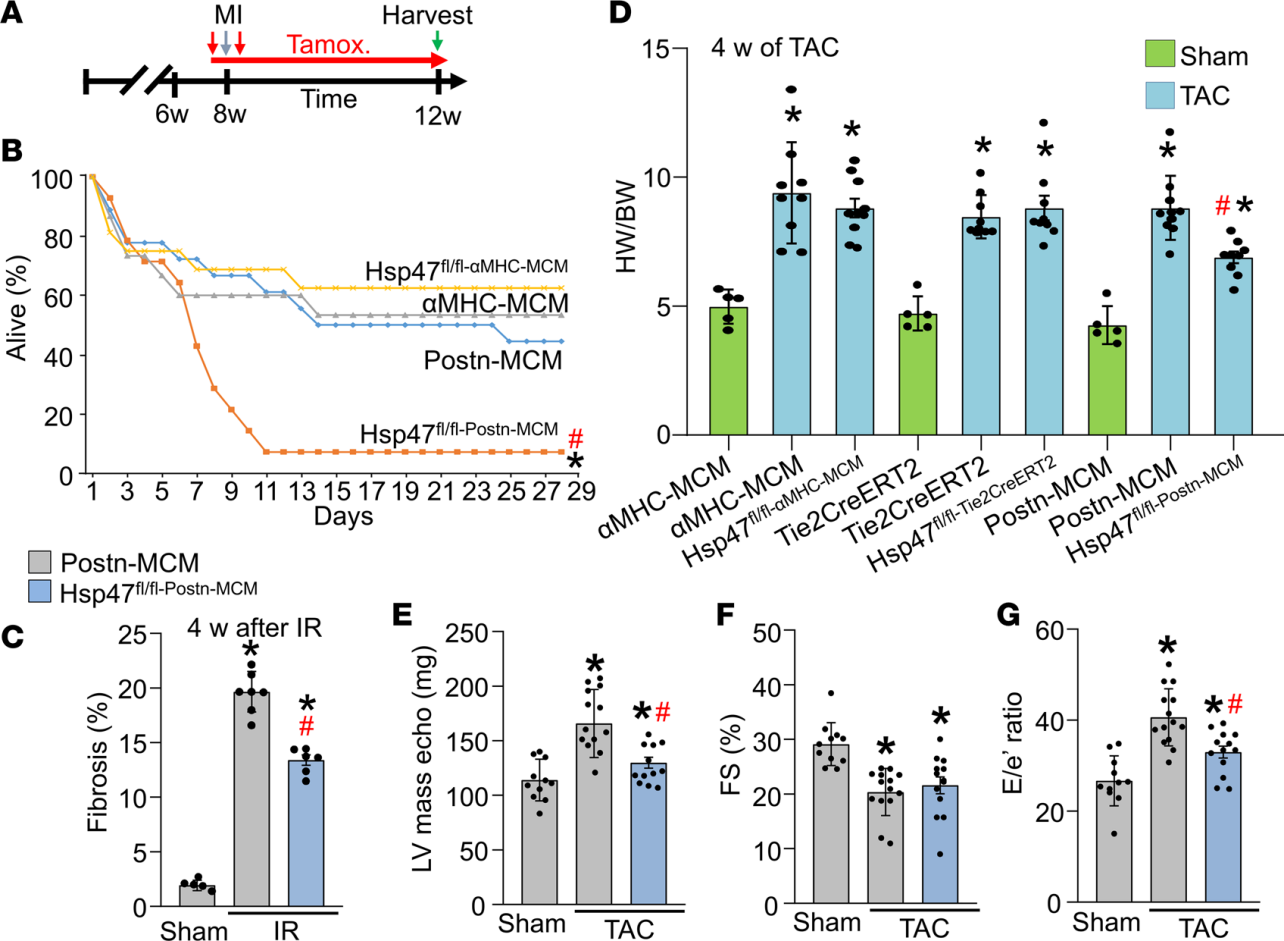
Myofibroblast-specific *Hsp47* deletion alters acute scar formation and the hypertrophic response. (**A**) Experimental scheme whereby αMHC-MerCreMer–transgenic mice or *Postn-MerCreMer* allele–containing mice were subjected to myocardial infarction injury for 4 weeks with 2 injections (vertical red arrows) of tamoxifen treatment and then tamoxifen in the feed for 4 weeks (horizontal red arrow). (**B**) Kaplan-Meier plot of survival of the indicated genotypes of mice after MI injury. *n* = 11–13 mice in each group. (**C**) Quantitation of fibrosis from Picrosirius red–stained histological sections from hearts after 4 week of I/R injury of the indicated genotypes. *n* = 6 mice in each group. (**D**) Gravimetric assessed heart-weight-to-body-weight (HW/BW) ratios in mice of the indicated genotypes after 4 weeks of TAC. *n* = 5 sham mice, *n* = 9–10 TAC mice in each group. *P < 0.05 versus sham; ^#^*P* < 0.05 versus *Postn-MCM* TAC. *P* values were calculated by 2-way ANOVA and Bonferroni post hoc test. (**E**–**G**) Echocardiographic assessment of ventricular (LV) calculated mass, left ventricular fractional shortening (FS%) percentage, and early mitral inflow velocity to mitral annular early diastolic velocity ratio (E/e) in the indicated genotypes of mice after 4 weeks of TAC injury or a sham procedure. *P < 0.05 versus *Postn-MCM* sham. ^#^*P* < 0.05 versus *Postn-MCM* TAC. *P* values were calculated with 1-way ANOVA with Tukey’s post hoc test. Number of mice used is shown in the scatter plots.

The mouse model of muscular dystrophy and the cardiac pressure overload surgical model represent chronic disease states whereby fibrosis slowly but progressively accumulates. In these long-term profiles the activated fibroblast was the primary mediator of the fibrotic response, but it is not clear if the myofibroblast is also the primary regulator of more acute injury where scar formation must occur quickly, such as after MI. To address this question, 8-week-old mice were given tamoxifen and then MI surgery was performed, after which mice were followed for survival to monitor the success of acute scar formation (Figure 5A). Myofibroblast-specific *Hsp47*-deleted mice were significantly compromised in their survival in the first week following MI injury, whereas all control groups and mice with cardiomyocyte-specific deletion of *Hsp47* (αMHC-MCM transgene) showed the typical low level of lethality with good overall survival and scar formation (Figure 5B). Upon postmortem inspection, the activated fibroblast-specific *Hsp47*-deleted mice uniformly showed left ventricular wall rupture, indicating that acute scar formation in the first week was compromised (data not shown). Thus, endogenous cardiac myofibroblasts are the primary cell type mediating both chronic disease-based fibrosis as well as acute healing and scar formation in the heart after MI injury. Since the standard MI injury induced close to 100% lethality of *Hsp47^fl/fl^Postn^MCM^* mice, we subjected these mice and their controls to an ischemia/reperfusion (I/R) procedure. The I/R procedure was better tolerated by *Hsp47^fl/fl^Postn^MCM^* mice, resulting in better survival rates. Cardiac sections were then quantified for the accumulation of cardiac fibrosis, which showed that surviving *Hsp47^fl/fl^Postn^MCM^* mice had a significant reduction in ventricular fibrotic area compared with *Postn^MCM^* controls (Figure 5C).

As part of our analysis we also measured the cardiac hypertrophic response by gravimetric analysis after 4 weeks of TAC-induced pressure overload stimulation. The data showed that deletion of *Hsp47* in cardiomyocytes or endothelial cells did not alter the otherwise robust development cardiac hypertrophy after pressure overload stimulation, yet *Postn*-MCM mediated deletion of *Hsp47* from myofibroblasts resulted in a significant reduction in cardiac hypertrophy versus controls (Figure 5D). Supporting these findings, myofibroblast-specific *Hsp47*-deleted mice also showed reduced cardiac hypertrophy after TAC compared with control mice, as assessed by echocardiography (Figure 5E). Cardiomyocyte area measured in histological sections and induction of hypertrophic marker genes were also reduced in the hearts of myofibroblast-specific *Hsp47*-deleted mice after TAC compared with controls (Supplemental Figure 7, A–D). Additionally, we evaluated the progress of myocardial hypertrophy in these mice and their controls after a shorter 10-day protocol of pressure overload, which once again showed a significant reduction in cardiac hypertrophy in myofibroblast-specific *Hsp47*-deleted mice (Supplemental Figure 7E). These results suggest that a deficiency in the ability of cardiac myofibroblasts to produce collagen with ECM accumulation reduces the cardiac hypertrophic response with pressure overload stimulation (see discussion).

Systolic functional performance of the heart remained equally compromised in myofibroblast-specific Hsp47-deleted mice compared with controls, as measured by echocardiography (Figure 5F), although diastolic performance measured indirectly by tissue Doppler echocardiography indicated a lower E/e′ ratio in myofibroblast-specific *Hsp47*-deleted mice after TAC relative to control, suggesting that reduced fibrosis attenuated TAC-induced diastolic dysfunction (Figure 5G).

### Deletion of Hsp47 reduces myofibroblast proliferation in vivo.

**Figure 6:**
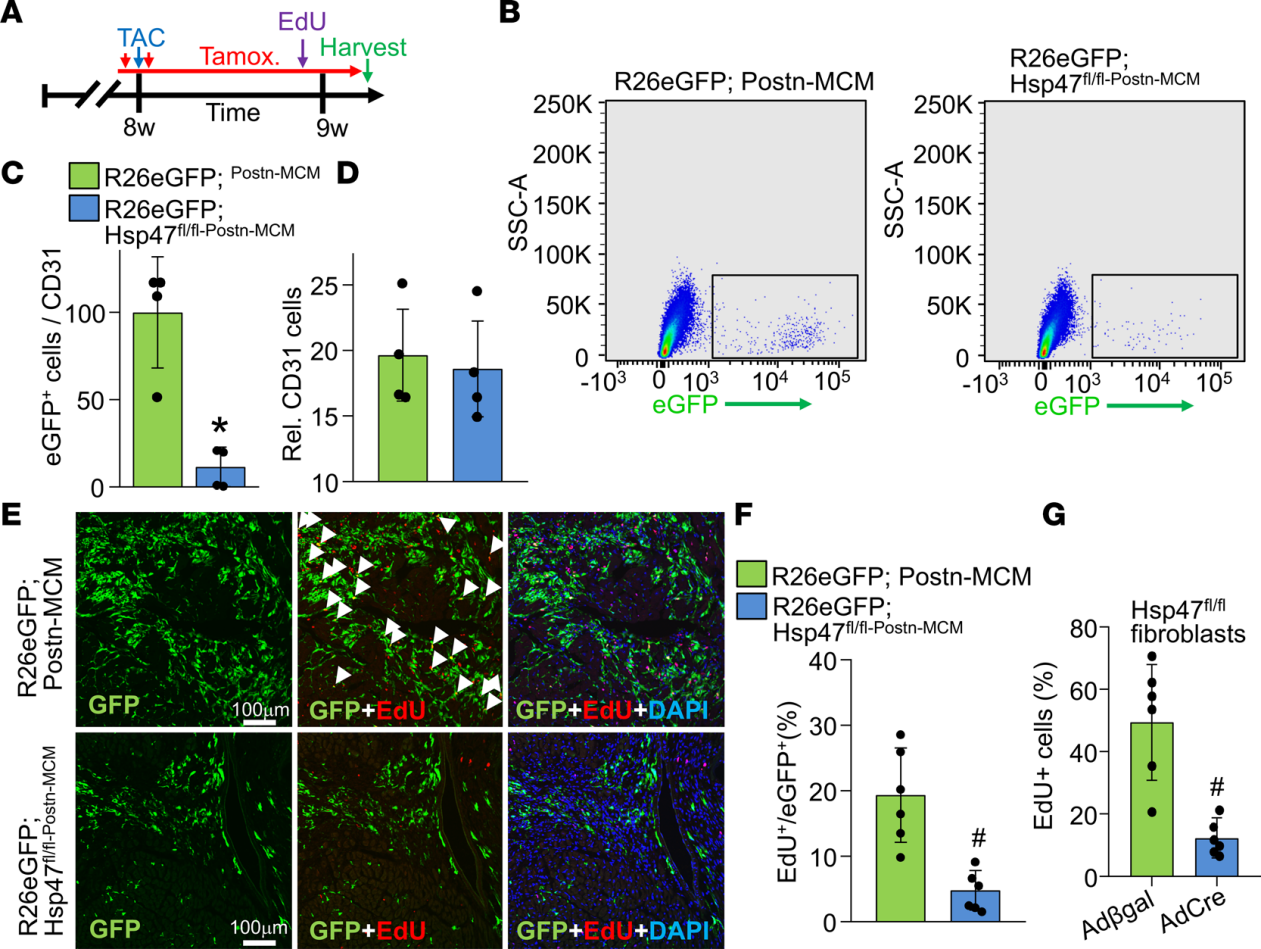
Myofibroblast-specific *Hsp47* deletion reduces myofibroblasts in vivo and their proliferation. (**A**) Experimental scheme whereby mice were subjected to TAC injury for 7 days. Mice received 2 i.p injections of tamoxifen and were fed tamoxifen-laden chow 48 hours before surgery and were then maintained on this chow until harvesting. Mice also received a single i.p EdU injection 4 hours before sacrifice at day 7 after TAC. (**B**) Representative flow cytometry plots of isolated EGFP^+^ interstitial cells (plotted as EGFP fluorescence signal on the *x* axis versus side scatter on the *y* axis) from hearts of the indicated genotypes of mice; 100,000 cells are displayed in the blots. (**C**) The ratio of total EGFP^+^ myofibroblasts normalized to CD31^+^ cells from the hearts of the indicated genotypes of mice after 1 week of TAC. Error bars represent SEM; *n* = 4 mice in each group. *P < 0.05 versus *Postn-MCM*; *R26^eGFP^*. *P* values were calculated with a Student’s *t* test. (**D**) Relative number of CD31^+^ cells in the interstitial fractions in hearts of the indicated genotypes of mice after 1 week of TAC. (**E**) Representative immunohistological images (scale bar: 100 μm) of EdU^+^ and EGFP^+^ interstitial cells at the time of harvest for mice treated, as shown in **A**. DAPI was used to show nuclei (blue). *n* = 5 mice in each group. (**F**) Quantitation of GFP^+^ cells that were also EdU^+^ in heart histological sections from mice subjected to TAC of the indicated genotype. *P* values were calculated with Student’s *t* test. ^#^*P* < 0.05 versus R26eGFP Postn-MCM controls. (**G**) Quantitation of EdU^+^ *Hsp47^fl/fl^* cardiac fibroblasts over 24 hours in culture previously treated with AdCre or Adβgal infection. A total of 6 images were analyzed per group. *P* values were calculated with Student’s *t* test. ^#^*P* < 0.05 versus Adβgal infection. Data shown are the mean ± SEM.

Deletion of *Hsp47* from myofibroblasts in the hearts of mice subjected to TAC significantly reduced production of the major fibrillar collagens and the fibrotic response, although the cellular basis underlying this effect was not clear. Here, we investigated the change in cardiac fibroblast dynamics over 1 week of TAC stimulation with or without *Hsp47* deletion in myofibroblasts (Figure 6A). To analyze fibroblast proliferation rates, mice of the genotype *Postn^MCM/+^Hsp47^fl/fl^R26e^GFP/+^* were compared with controls of the genotype *Postn^MCM/+^R26e^GFP/+^*. Mice were also given 5-ethynyl-2′-deoxyuridine (EdU) treatment to measure proliferation, which was given in the last 4 hours before sacrifice (Figure 6A). Hearts were removed and total interstitial cells were isolated after enzymatic digestion followed by FACS to quantify total EGFP^+^ cells normalized to CD31^+^ endothelial cells (Figure 6, B and C). Endothelial cells expanded similarly with pressure overload stimulation in the 2 experimental groups with or without *Hsp47* in activated cardiac fibroblasts (Figure 6D). The data show a dramatic reduction in total numbers of activated EGFP^+^ myofibroblasts in the hearts of *Hsp47*-deleted mice after 1 week of TAC compared with control mice (Figure 6, B and C). To investigate whether the reduction of total activated fibroblasts is due to a decrease of proliferation rate, histological sections were quantified for EGFP^+^ fibroblasts and for EdU incorporation (Figure 6, E and F). After 4 hours of EdU exposure, about 20% of EGFP^+^ activated fibroblasts incorporated EdU suggesting they were in cell cycle, while less than 5% of the *Hsp47*-deleted EGFP^+^ myofibroblasts incorporated EdU (Figure 6, E and F). We also assessed cell death of myofibroblasts by TUNEL, which showed that myofibroblast-specific Hsp47-deletion produced a significant increase in TUNEL^+^ myofibroblasts compared with control (Supplemental Figure 1B).

To extend these results, primary *Hsp47^fl/fl^* cardiac fibroblasts were isolated and infected with AdCre or Adβgal for 72 hours to evaluate cellular proliferation using (EdU) over 24 hours. Approximately 55% of Adβgal infected fibroblasts incorporated EdU, while only approximately 15% of the AdCre-infected fibroblasts were proliferative (Figure 6G). Thus, loss of *Hsp47* from myofibroblasts in the heart compromises their proliferation with TAC stimulation, suggesting an additional contributing factor to the observed reduction in fibrosis and cardiac hypertrophy. Indeed, we have previously shown that fibroblasts normally undergo a 3-fold expansion with injury to the heart that is associated their ability to effectively generate fibrosis and a scar (47).

### Gene expression profiling defines a reduced collagen and differentiated state of Hsp47-deleted myofibroblasts.

**Figure 7:**
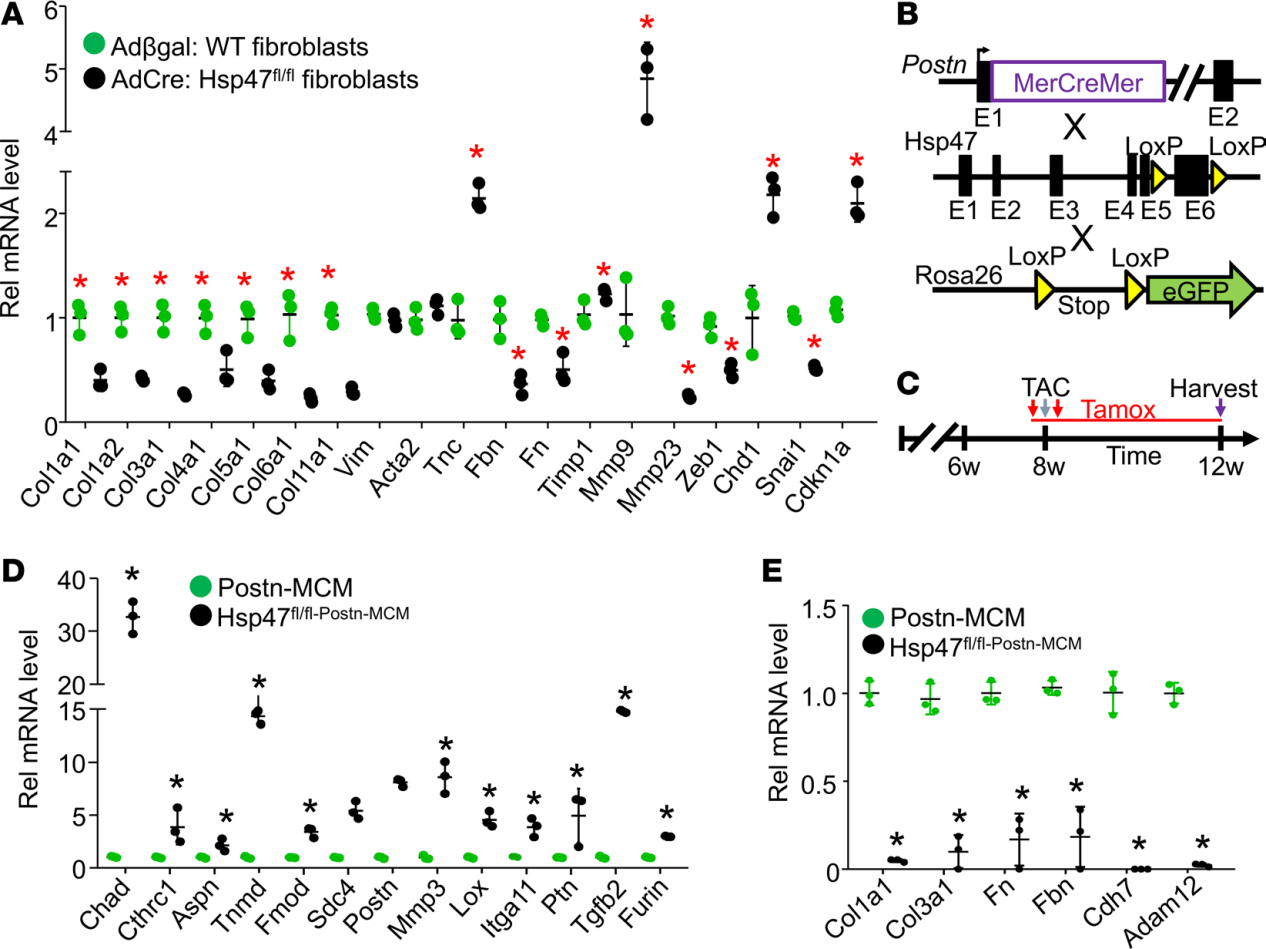
*Hsp47* deletion in myofibroblasts reduces ECM-related gene expression and promotes an altered differentiated state. (**A**) Adult primary heart fibroblasts were isolated from *Hsp47-loxP*–targeted mice infected with Adβgal (WT) or AdCre (deleted samples). Seventy-two hours after infection cells were washed and incubated in 2% serum containing DMEM media with 20 μM ascorbic acid for 24 hours before RNA isolation. The data are real-time PCR results showing the expression levels of the indicated genes. *n* = 4 separate experiments. *P < 0.05 versus Adβgal WT. (**B**) Schematic representation of the *Postn-MCM* mouse line crossed with the *Hsp47-loxP* site–containing gene–targeted line and the *Rosa26* reporter line (*R26^eGFP^*). (**C**) Experimental scheme with TAC stimulation and tamoxifen with injection and laden food. (**D** and **E**) Quantification of selected mRNAs in *Hsp47*-deleted EGFP^+^ myofibroblasts isolated from hearts of *Hsp47^fl/flPostn-MCM/+^R26^eGFP/+^* allele containing mice, 4 weeks after TAC injury. *n* = 3, *P < 0.5. *P* values were calculated with Student’s t test. Data shown are the mean ± SEM.

Here we instituted mRNA profiling in *Hsp47*-deleted myofibroblasts to gain insight into the molecular alterations that might underlie their reduced activity and proliferation. We first assessed mRNA levels of endogenous *Col1a1*, *Col3a1*, *Col4a1*, *Col5a1*, *Col6a1*, and *Col11a1* from primary *Hsp47^fl/fl^* cardiac fibroblasts in culture infected with AdCre or Adβgal. Unexpectedly, the data showed a generalized and significant reduction in mRNA expression of all the collagen genes tested with *Hsp47* deletion (Figure 7A). In parallel, we assessed the expression profile of a panel of ECM-related genes, including fibrillin (*Fbn*), fibronectin *(Fn*), and *Mmp23,* which were also downregulated in *Hsp47*-deleted fibroblasts. By comparison, we observed induced expression of tenascin-C (*Tnc*) and *Mmp9*. Interestingly, loss of *Hsp47* in fibroblasts did not affect expression of smooth muscle α-actin (*Acta2*) versus WT control fibroblasts (Figure 7A). In addition, *Hsp47*-deleted fibroblasts had diminished replicative capacity in culture, which is consistent with the observed downregulated expression of zinc finger E-box–binding homeobox transcription factor 1 (*Zeb1*) and zinc finger transcription factor (*SnaiI*), and increased expression of the cell cycle inhibitory cyclin–dependent kinase inhibitor, Cdkn1a (Figure 7A).

We also performed unbiased and global mRNA profiling of FACS-isolated EGFP^+^ activated myofibroblasts from the heart after 4 weeks of pressure overload, with and without *Hsp47* deletion, as shown in Figure 7, B and C. Bioinformatics analysis identified modulation in genes implicated in key functional features of the fibroblast, such as ECM components, ECM modification, bone and cartilage signatures, proliferation, cell adhesion, and TGF-β signaling (Supplemental Table 1). The most notable upregulated genes identified in *Hsp47-*deleted myofibroblasts were those underlying bone, cartilage, and tendon development or processing, followed thereafter by ECM-associated proteoglycan genes (Supplemental Table 1). Genes regulating cell adhesion and TGF-β signaling were also identified as significantly altered with *Hsp47* deletion after TAC stimulation (Supplemental Table 1). Collectively, the bioinformatics profile showed that loss of *Hsp47* reduced the expression of ECM-related genes, reduced expression of proliferation genes, and showed mRNA signatures that are consistent with premature senescence.

In parallel, the expression profile of representative gene categories was confirmed by RT-PCR (Figure 7, D and E). Consistent with the previous data obtained in primary cardiac fibroblasts in culture, the data from in vivo isolated EGFP^+^ myofibroblasts showed that loss of *Hsp47* caused a generalized and significant reduction in expression of mRNA levels of endogenous *Col1a1*, *Col3a1*, *Fn*, *Fbn*, and *Adam12* (Figure 7E). Similarly, an induction of cartilage associated chondroadherin (*Chad*), leucine-rich repeat protein asporin (*Aspn*), tenomodulin (*Tnmd*), and the proteoglycans fibromodulin (*Fmod*) and syndecan-4 (*Sdc4*) was observed in *Hsp47-*deleted myofibroblasts, which could alter the adhesive state of these cells (Figure 7D). Modulated mRNA expression levels of cell surface receptors, including integrin subunit α 11 (*Itga11*) and cadherin-7 (*Cdh7*) were also identified, and we confirmed an induction in the mRNA levels of TGF-β signaling genes (*Tgfb2* and *Furin*) and the gene for TGF-β–inducible glycoprotein collagen triple helix repeat-containing protein 1 (*Cthrc1*) that is known to reduce collagen type I mRNA (48). Finally, the expression levels of genes integral for ECM organization and maturation, such as lysyl oxidase (*Lox*), *Mmp3*, and *Postn* were found to be induced in *Hsp47-*deleted myofibroblasts (Figure 7D).

## Discussion

ECM in the adult heart is composed predominantly of fibrillar collagens in addition to matricellular proteins, proteoglycans, and growth factors (2, 3). Fibrillar collagens within the ECM microenvironment serve as a critical component in supporting cardiomyocyte contraction and possibly even stretch-regulated reactive signaling (4). Collagen deposition is often upregulated in diseased hearts, which can cause an irreversible and chronic fibrotic profile that increases ventricular stiffness and reduces contractile dynamic range (49, 50). While it is accepted that myofibroblasts express and secrete collagens and other ECM proteins, so do cardiomyocytes (34, 35, 51–53) and endothelial cells (31–33). The results presented here are the first to our knowledge to directly demonstrate in vivo that it is the myofibroblast that serves as the primary source of collagen production in the acutely stressed or chronically diseased heart. Moreover, deletion of *Hsp47* within myofibroblasts of skeletal muscle, but not within the myofibers, also reduced the extent of muscular dystrophy disease–dependent fibrosis, suggesting that it is the myofibroblast that is the primary regulatory of adult disease-based tissue fibrosis in general. However, we did observe a mild, albeit significant reduction in collagen type I expression in the heart after endothelial cell–specific deletion of *Hsp47*, suggesting that endothelial cells may still play a minor role in cardiac fibrosis with pressure overload stimulation. The results observed here are also consistent with past data, whereby selective inhibition of key signaling pathways in myofibroblasts can disrupt the greater fibrotic response of the heart (54–57). For example, we previously showed that myofibroblast-specific deletion of *Smad2* and *Smad3* or *Tgfbr1* and *Tgfbr2* reduced cardiac fibrosis in the heart during pressure overload stimulation by compromising the activity of the TGF-β signaling pathway in myofibroblasts (55).

Four weeks of pressure overload stimulation in mice typically results in reduced cardiac function, such as a reduction in ventricular fractional shortening, as measured by echocardiography (Figure 5C). Interestingly, the reduction in myocardial fibrosis in myofibroblast-specific *Hsp47-*deleted mice did not protect from cardiac decompensation compared with control mice, suggesting that fibrosis in the first 4 weeks of pressure overload is not a primary inducer of decompensation. However, the reduced ECM profile associated with *Hsp47* deletion in myofibroblasts did reduce the extent of cardiac hypertrophy over 4 weeks of pressure overload, suggesting that greater ECM content is needed to support fulminant hypertrophic growth of cardiomyocytes. Alternatively, it is also possible that the failure of fibroblasts to expand renders the heart with fewer fibroblast-dependent paracrine factors to support cardiomyocyte growth. Similarly, we previously observed that deletion of *Tgfbr1/2* in activated fibroblasts of the mouse heart during TAC stimulation lead to a reduction in cardiomyocyte hypertrophy, and fibroblasts with *Tgfbr1/2* deletion were again less active (55, 57). However, while our results do not allow us to discern the exact mechanism whereby a compromised cardiac fibrotic response renders the heart significantly less hypertrophic, we favor the interpretation that augmented ECM content and structural support are required for optimal growth of cardiomyocytes as the dominant mechanism in play. Indirect support for this hypothesis comes from cardiac development itself, whereby myocytes within the neonatal heart begin to hypertrophy to their adult state in coordination with a switch in the cardiac ECM from fibronectin to a stiffer and more structurally supportive collagen type I platform (58).

Unexpectedly, loss of collagen production in *Hsp47-deleted* myofibroblasts induced a negative feedback loop resulting in the reduction of mRNA expression of multiple collagens (*Col1a1*, *Col2a1*, *Col3a1*, *Col4a1*, *Col5a1*, *Col6a1*, and *Col11a1*). In parallel *Hsp47*-deleted myofibroblasts seemed to initiate a compensatory mechanism that includes the induction of matricellular proteins and proteoglycans, such as *Tnc*, *Postn*, *Sdc4*, and *Fmod* as well as the glycoprotein *Tnmd*. *Hsp47*-deleted myofibroblasts also showed an altered differentiated state with increased expression of *Chad* and *Aspn* (47, 59). We have previously shown that deletion of the *Postn* gene, which is a matricellular protein that participates in collagen maturation, similarly alters the differentiated state of myofibroblasts from the heart and leads to an attenuated fibrotic response with injury (60). Similarly, in lung fibroblasts, knockdown of select lysyl oxidases that are required for proper extracellular collagen maturation changes the expression of select fibroblast functional genes (61). Thus, the content and relative abundance of ECM and collagen within the heart are critical regulators of fibroblast biology and ECM-related mRNA levels through some sort of feedback regulation. However, as stated above, it is not clear how this regulation occurs; it is possibly through paracrine or autocrine factors that are stored within the ECM and released as signals, or the structural properties of the ECM itself support fibroblast activity and differentiation through direct adhesion complexes and their inherent intracellular transducing properties.

The observations presented here suggest an important regulatory relationship between different cardiac cell types within the heart and the ECM microenvironment. Both cardiomyocytes and fibroblasts appear to directly sense the extent of ECM construction in the heart with acute or chronic injury. Indeed, with an inability of fibroblasts to expand the ECM during pressure overload, the cardiomyocytes cannot attain the same degree of hypertrophy. At the same time, newly activated fibroblasts show a defect in proliferation and an altered differentiated gene program (more senescent). Thus, while our results proved the longstanding hypothesis that it is the myofibroblast underling cardiac disease-based fibrosis in vivo, we uncovered an unanticipated regulatory feedback relationship between the collagen content in the heart and hypertrophic growth potential of cardiomyocytes and activity of fibroblasts themselves. These results suggest some obvious clinical vantage points, such as simply inhibiting collagen production or maturation in the ECM as an antifibrotic strategy in heart failure or muscular dystrophy.

## Methods

### Mice.

Mice containing a genetic insertion of a tamoxifen-inducible MCM cDNA, into the *Postn* locus, or transgenic αMHC-MCM mice, were used and described previously (36, 38). *Rosa26* loxP site–dependent reporter mice (*R26^eGFP^*) were previously described (62) and were purchased from the Jackson Laboratories (stock no. 012429). βMHC-Cre mice were described elsewhere (37), as were skeletal α-actin MCM-transgenic mice (46). A mouse model of limb-girdle muscular dystrophy lacking the δ-sarcoglycan gene (*Sgcd^–/–^*) was also used (43). Tie2-CreERT2–transgenic mice were described elsewhere (40), as were loxP site–targeted *Hsp47* mice (28). PCR genotyping used the following primers, which generated a 450-bp product recognizing loxP-targeted exon 6 and 350-bp WT fragments: Hsp47 forward 5′-GAGTGGGCTGAGCCCTCTCAAGAAAATCC-3′ and reverse 5′-CTTCGGTCAGGCCCAGTCCTGCCAGATG-3′. Mice received a combination of tamoxifen citrate chow (400 mg/kg body weight, Envigo-TD, 130860) and i.p. injections with pharmaceutical-grade tamoxifen (75 mg/kg body weight, MilliporeSigma, T5648) dissolved in 95% corn oil/ 5% ethanol. The *Postn-MCM* line was i.p. injected once 48 hours before surgery and once 24 hours after surgery and maintained on tamoxifen citrate chow 48 hours before surgery for the entirety of the experiment. The Tie2-CreERT2–transgenic mouse line received 2 i.p. injections with tamoxifen 15 days before the surgical procedure followed by feeding with tamoxifen citrate chow 48 hours before surgery until the experiment was terminated. The αMHC-MCM–transgenic line received 3 i.p. injections on 3 consecutive days with pharmaceutical-grade tamoxifen 2 weeks before the surgical procedure. Mice in the *Sgcd^–/–^* background received tamoxifen in the chow beginning at 1 month of age until the experiment was terminated. All mice were in the C57BL/6 genetic background, and male mice were used throughout to reduce the total number of animals needed to achieve statistical significance and to reduce variation.

### Animal procedures.

All experimental procedures with mice were approved by the Institutional Animal Care and Use Committee of Cincinnati Children’s Medical Center, protocols IACUC 2015-0047 and 2016-0069. The number of mice used in this study reflects the minimum number needed to achieve statistical significance based on experience and previous power analysis. Blinding was performed for some experimental procedures with mice, although blinding was not possible in every instance. Randomization of mouse groups was not performed because mice were genetically identical and often littermates, although only males were used to reduce total animal usage and to limit variability. Eight-week-old mice were subjected to cardiac pressure overload by TAC surgery as described previously (55). Echocardiography was performed in a blinded fashion to assess ventricular geometry and function at 4 weeks after TAC as described previously (55). Briefly, animals were anesthetized with 2% isoflurane inhalation and analyzed with a Vevo 2100 instrument equipped with 18- to 38-MHz transducer (VisualSonics). Fractional shortening, left ventricular end-diastolic volume, and left ventricular mass were determined from 2D M-mode echocardiograms. The effectiveness of the TAC procedure was verified by Doppler echocardiography, which measured pressure gradients across the aortic constriction. Diastolic function was evaluated by tissue Doppler echocardiography presented as the E/e′ ratio that measures the mitral valve inflow maximum velocity (E-wave) to posterior wall maximum tissue Doppler velocity (e′) ratio. MI was induced in mice via permanent surgical ligation of the left coronary artery (63). Briefly, 2% isoflurane–anesthetized mice were subjected to a left lateral thoracotomy and the left coronary artery was isolated and occluded permanently just below the left atrium. I/R injury was described previously (64). Briefly, mice were anesthetized with inhaled 2% isoflurane, intubated through the mouth, and ventilated throughout the procedure. I/R injury was induced with a slipknot around the left coronary artery. After 30 minutes of ischemia, the slipknot was released followed by reperfusion until the mice were euthanized and the hearts harvested. A single postoperative dosage of buprenorphine at 0.1 mg/kg was given by subcutaneous injection to reduce pain.

### Histology and immunostaining.

Isolated hearts were fixed for 4 hours in freshly diluted 4% paraformaldehyde at 4°C, and then a portion of the tissue was rinsed with PBS and cryoprotected in 30% sucrose/PBS overnight before embedding in OCT (Tissue-Tek). Another part of the tissue was washed with 70% ethanol and subjected to paraffin embedding. Afterward, 10-μm cryosections were collected and then processed for 30 minutes at room temperature in a blocking solution (PBS with 5% goat serum, 2% bovine serum albumin, 0.1%Triton X-100). Collagen type I, collagen type III, and collagen type V primary antibodies (Abcam ab21286, ab7778, and ab7046, respectively, 1:100 dilution) were diluted in blocking solution and incubated on the histological sections overnight at 4°C. The sections were then washed 3 times for 5 minutes each in PBS and incubated with Alexa Fluor 568–conjugated goat anti-mouse antibody (Life Technologies, A11031) for 2 hours at room temperature at 1:400 dilutions. After washing 3 times for 5 minutes each, sections were stained with DAPI at a concentration of 0.1 μg/ml in water for 5 minutes at room temperature and mounted on slides using aqueous mounting medium (H-1400, Vector Laboratories). In some experiments, cryosections were used to visualize native EGFP fluorescence from the appropriate genotypes that contained the *Rosa26*-loxP–dependent reporter allele. Images were acquired with an inverted Nikon A1R confocal microscope using NIS Elements AR 4.13 software. Ten random pictures per mouse heart were taken from 5–10 sections each at different levels of the heart for quantitative analysis. Quantification of signal intensity was done by calculating pixels numbers using with Adobe Photoshop Elements 9.

### Picrosirius red staining and hydroxyproline assessment.

Picrosirius red staining was done with a kit (Electron Microscopy Sciences, kit 26357-02) per manufacturer’s instructions. Picrosirius red images were captured with a Leica M165FC stereo microscope with fluorescence using a Leica DFC310 FX camera and the Leica Application Suite. Total collagen content in cultured media was analyzed by using the Sirius Red Total Collagen Detection Kit (Chondrex, 9062). Hydroxyproline content in cardiac tissue was assayed as detailed previously (65).

CHP staining was described earlier (39) and was purchased from 3Helix (Bio300). Briefly, 4% paraformaldehyde-fixed tissue sections were blocked with 2 drops of the streptavidin and biotin reagent according to the manufacturer protocol for 15–30 minutes at 37°C in a humid chamber (Thermo Fisher Scientific, E21390). Slides were rinsed twice with PBS for 10 minutes. Sections were then blocked with PBS supplemented with 0.2% Triton X-100, 5% goat serum, and 1% BSA and incubated for 30 minutes at room temperature. A 15 μM CHP working solution was freshly prepared and warmed at 80°C for 5 minutes and then instantly placed on ice for 15 seconds and applied to the histological sections on glass slides. The exact procedure was followed when CHP was used with cells on coverslips; however, the final concentration of company-supplied reagent was 3 μM. The slides were then incubated at 4°C overnight and subsequently washed and incubated with a Alexa Flour 568–conjugated (Life Technologies, S11226) secondary streptavidin-conjugated antibody.

### Western blot.

Western blotting was performed as described previously (66). Briefly, protein preparations were mixed with 5× Laemmli loading buffer and heated to 95°C for 10 minutes. Equal amounts of protein were subjected to electrophoresis and transferred to polyvinylidene fluoride (PVDF) membranes (EMD Millipore, IPFL00010). The membranes were incubated with antibodies against Hsp47 (1:000, Novus Biologicals, M16.10A1), Gapdh (1:10000, Fitzgerald, 10R-G109a), or α-tubulin (1:1000, Santa Cruz, sc-8035). PVDF membranes were then incubated with the appropriate Alexa Fluor–conjugated secondary antibodies (Thermo Fisher Scientific, A21057, Goat anti-Mouse IgG Cross-Adsorbed Secondary Antibody, Alexa Fluor 680, 1:5,000; Thermo Fisher Scientific, A11367, Goat anti-Rabbit IgG Cross-Adsorbed Secondary Antibody, Alexa Fluor 790, 1:5000) and then visualized by using an Odyssey CLx imaging system (LI-COR Biosciences, 9140).

### Adult cardiomyocyte and interstitial heart cell isolation and ECM protein preparations.

Adult cardiomyocytes and interstitial heart cell fractions were isolated as described previously (55). Briefly, freshly beating hearts were cannulated for retrograde perfusion with modified Tyrode solution (120 mM NaCl, 14.7 mM KCl, 0.6 mM KH_2_PO_4_, 0.6 mM Na_2_HPO_4_, 1.2 mM MgSO_4_, 10 mM HEPES, 4.6 mM NaHCO_3_, 30 mM taurine, 5.5 mM glucose, and 10 mM butanedionemonoxime, pH 7.4), supplemented with liberaseTH (Roche, 05401151001). Hearts were then dissociated into cardiomyocytes and noncardiomyocyte fractions and then separated by 2 serial centrifugations at 10 *g* for 5 minutes at 4°C. The noncardiomyocyte cell fraction was centrifuged at 500 *g* for 10 minutes at 4°C. ECM protein preparations were performed as previously described (67).

### Isolation of adult cardiac fibroblasts and endothelial cells.

Cardiac ventricles were excised from mice, rinsed with cold sterile 1× Hanks’ Balanced Salt Solution (HBSS, Gibco, 14025092) and treated as described previously (68). Briefly, tissues were thoroughly minced with sterile fine scissors and digested in 2 ml DMEM containing a combination of 2 mg/ml collagenase type IV (Worthington, LS004188) and 0.75 mg/ml dispase II (Roche, 10165859001) at 37°C for 60 minutes (20-minute incubation). During incubations, the digesting tissue was triturated for a minute with a sterile serological pipette every 15 minutes, and the digestion mix was incubated for 2 minutes to sediment before collecting the supernatants. The supernatant cell suspension containing the liberated fibroblasts was then collected in a tube containing cold DMEM supplemented with 10% bovine growth serum (BGS) (GE Healthcare Life Sciences, SH30541.03). The undigested fraction was reconstituted with 2 ml fresh digestion media, and the same digestion procedure was repeated for 3 total rounds. Cell debris was eliminated by 2 serial centrifugations at 10 *g* for 5 minutes at 4°C, and the cell fraction was collected after a final centrifugation at 500 *g* for 10 minutes at 4°C. For flow cytometry analysis, pellets were washed once with ice-cold HBSS incubated for 1 minute in 1 ml red blood cell lysis buffer (MilliporeSigma, R7757) and then washed, centrifuged, and resuspended in 2% BGS and 2 mM EDTA in HBSS and incubated on ice for FACS analysis using a Becton Dickinson Aria Instrument (a BD FACSAria III). A fraction of this preparation was cultured in 10% BGS containing DMEM media on gelatin 0.1% coated plates for further in vitro analysis.

### Flow cytometry and cell sorting.

Flow cytometry analysis was performed on isolated cardiac interstitial cells using a BD FACSCanto II running FACSDiva software with the following configuration: 405 nm laser for Alexa Fluor 405, 633 nm for APC, and 488 nm for EGFP. Analysis was performed using FlowJo vX (BD). To obtain endothelial cell counts, isolated cells were stained with APC-conjugated antibodies against CD31 (eBioscience, 17-0311-82) at a 1:200 dilution in 2% BGS in HBSS incubated for 30 minutes on ice. At the end of the incubation time, cells were washed 3 times and analyzed. For sorting and analysis of lineage-traced genotypes, we utilized the endogenous EGFP fluorescence expressed by the recombined *R26^eGFP^* reporter allele due to the activity of the Tie2-CreERT2 transgene and *Postn-MCM* allele. The count of EGFP^+^ cells was normalized to the number of CD31^+^ cells to control for the degree of variability in sample digestion and cellular isolation.

### Fibroblast cultures and treatments.

Adult heart fibroblasts were cultured in DMEM (Fisher Scientific, SH30022FS) supplemented with 10% BGS and nonessential amino acids. Depending on the experiment, cultured fibroblasts were infected with a Cre recombinase–expressing adenovirus (AdCre) and compared with a control Adβgal-expressing adenovirus. The cells were incubated with these recombinant adenoviruses for 4 hours in serum-free media and then washed and maintained in DMEM media for 72 hours. Collagen synthesis was induced by treatment with 100 μM ascorbic acid (MilliporeSigma, A5960) and/or 10 ng/ml TGF-β (R&D Systems, 101-b1-010) over 24 hours in serum-free DMEM media.

### Neonatal cultures for stretching experiments.

Neonatal rat cardiomyocytes and fibroblasts were isolated from 1- to 2-day-old rat pups as described previously (69). Ventricles were placed in an isotonic salt solution composed of 116 mM NaCl, 5.4 mM KCl, 20 mM HEPES, 0.9 μM Na_2_HPO_4_, 5.4 mM MgSO_4_, and 5 mM glucose. The ventricles were then enzymatically digested using 84 U/ml collagenase type I (Worthington, LS005273) and trypsin (Worthington, LS003736). After isolation, cardiomyocytes were cultured overnight in M199 medium (Mediatech, 10-060-CV) supplemented with 15% FBS, penicillin/streptomycin (100 U/ml), and l-glutamine (2 mM). The following day, the medium was replaced and cardiomyocytes were cultured in 1% serum-containing M199 medium. Cardiomyocytes were seeded at a preadherent density of 0.5–1.0 laminin-coated Flexel 6-well 2.5-cm culture dishes. Neonatal rat cardiac fibroblasts were cultured as described above and seeded on 0.01% coated gelatin Flexel plates (Flexcell International). Stretch-induced stress was performed after seeding cells on 6-well Flexcell culture plates and applying a 20% stretch to the membranes using the Flexcell vacuum device (28-mm posts, Flexcell International). Stretch oscillations were performed continuously for 24 hours at 1.0 Hz and then harvested for Western blotting.

### Secretome profiling of cultured cardiac fibroblasts.

To assess the secreted factor profile of cardiac fibroblasts with or without Hsp47, cells were isolated from *Hsp47-loxP* mice and infected with adenoviruses expressing either Cre recombinase or β-galactosidase as described earlier. Following 24 hours of culture in serum-free media, the media were removed, centrifuged at 14,000 *g* to remove cellular debris, and concentrated over Amicon Ultra-4 Centrifugal Filter Unit columns (EMD Millipore). Samples were then analyzed using the Proteome Profiler Mouse XL Cytokine Array (R&D Systems, ARY028) per the manufacturer’s instructions, except that a streptavidin-conjugated Alexa Fluor IRDye 800 (LI-COR Biosciences) was used for detection with an Odyssey CLx imaging system (LI-COR Biosciences). Quantitation was performed using LI-COR Image Studio v.3.1.4.

### RNA-sequencing and bioinformatics analysis.

RNA from EGFP^+^ fibroblasts was isolated with the miRNeasy Micro Kit (Qiagen, 217084). Total RNA amplification (NuGEN, 7102-32), library generation (Illumina Technologies, FC-131-1002), and cDNA fragmentation (Amplicon Tagment Mix, FC-131-1096) were described previously (55). The purified cDNA was captured on an Illumina flow cell for cluster generation. Libraries were sequenced on the Illumina HiSeq2500 instrument within the Human Genetics Department at Cincinnati Children’s Hospital. Bioinformatics analysis was carried out using AltAnalyze software (70) to identify differentially expressed genes. Gene cluster analysis of biologic pathway–based expression groupings was done using Gene Ontology Consortium enrichment analysis (71, 72). The RNA-sequencing data were deposited with the GEO database group and given accession number GSE129612. A set of modulated target genes observed in RNA-sequencing results was validated by quantitative reverse transcriptase PCR (qRT-PCR).

### qRT-PCR.

RNA was isolated from FACS-sorted cardiac tissue or isolated cells using a Qiashredder homogenization instrument (Qiagen, 79654) and the RNAeasy kit according to the manufacturer’s instructions (Qiagen, 217084). Total RNA was reverse transcribed using random oligo-dT primers with a Verso cDNA synthesis kit (Thermo Fisher Scientific, AB1453) according to manufacturer’s instructions. Quantitative real-time PCR was performed using Sso Advanced SYBR Green (Bio-Rad, 6090). ΔΔCT was used to quantify the fold change of the target genes, and Gapdh expression was used for normalization. The primer sets used to identify transcripts are presented in Supplemental Table 2.

### EdU staining in vivo and in vitro.

EdU was purchased from Life Technologies (E10187) and prepared as a 5 mg/ml stock solution in PBS; it was i.p. injected into mice at 50 mg/kg 4 hours before sacrifice. After isolation and fixation, heart sections were stained for EdU by using the Click-iT Plus EDU Alexa Fluor 647 imaging kit according to the manufacturer’s protocol (Thermo Fisher Scientific, C10340). Mouse cardiac fibroblasts were isolated as previously described and treated with either an adenoviral vector encoding β-galactosidase (control) or Cre (to delete *Hsp47*) in 2% BGS (BGS) supplemented DMEM. Three days later, cells were incubated for 24 hours with 10 μm 5EdU before fixation in 4% paraformaldehyde for 20 minutes. Samples were washed with PBS, and EdU^+^ cells were detected using the Click-iT EdU Alexa Fluor 647 Imaging kit. Cell nuclei were detected by incubation with DAPI for 15 minutes (1:5000, Thermo Fisher Scientific, D3571). Cells were washed and mounted with Prolong Diamond anti-fade (Thermo Fisher Scientific, P36965). Six images were randomly taken per group and analyzed in a blinded manner using NIS-elements advanced research software (Nikon).

### Treadmill running.

Mice were subjected to forced downhill treadmill running using a ramping speed protocol as previously described (73). Briefly, 4-month-old mice were acclimatized to a motorized treadmill apparatus (Columbus Instrument) for 5 minutes at 0 m/min followed by 3 minutes at 6 m/min without electrical stimulation. Subsequently, mice were subjected to a forced downhill (10 degrees) treadmill running protocol where the speed was increased in increments of 2 m/min every 3 minutes to a final speed of 18 m/min, during which the mice were subjected to a mild electrical shock (3 Hz) on a pad at the bottom of the treadmill to motivate their continue efforts.

### Statistics.

Two-way ANOVA with Bonferroni’s post hoc honestly significant difference analysis was used to determine statistical significance when comparisons were made across different Cre lines. One-way ANOVA with Tukey’s post hoc honestly significant difference analysis was used to determine statistical significance when comparisons were made within a single Cre line. Averaged data are presented with SEM to indicate variability. Survival curves (Kaplan-Meier plots) were compared by log rank test. *P* < 0.05 was considered statistically significant. Analyses were performed using Prism 8 software.

### Study approval.

Mice were observed daily, and cages were changed every 2 weeks by veterinary technicians at Cincinnati Children’s Hospital Medical Center. Mice were assessed for their well-being by noting adequate physical activity and food intake on a daily basis. Housing conditions and husbandry at Cincinnati Children’s Hospital conformed to AAALAC standards and the institution’s ongoing certification by this organization as well as by the standard guidelines from the Office of Laboratory Animal Welfare (OLAW, https://olaw.nih.gov/guidance/topic-index/animal-use.htm). All animal experimentation related to this study was approved by the Office of Research Compliance and Regulatory Affairs at Cincinnati Children’s Hospital through the Institutional Animal Care and Use Committee (protocol IACUC 2016-0069, expires 11-2019). No human subjects were used.

## Author contributions

HK, OK, AKJ, BDM, RJV, VP, JGB, MJB, TS, KKK, and RNC conducted experimentation. HK designed and conducted experiments and acquired and analyzed data. KN and KK generated the *Hsp47*-loxP–targeted mice. JDM and HK conceived of the study, directed the study, and wrote the manuscript.

## Supplementary Material

Supplemental data

## Version 1 07/02/2019

In-Press Preview

## Version 2 08/08/2019

Electronic publication
