# Supplementary material for: Cell-specific ablation of Hsp47 defines the collagen-producing cells in the injured heart
Source: JCI Insight. 2019 Aug 8;4(15):e128722. doi: 10.1172/jci.insight.128722 (PMC6693833; doi:10.1172/jci.insight.128722)
Supplement: Supplemental data [file jciinsight-4-128722-s067.pdf]

Cell-specific ablation of Hsp47 defines the collagen producing cells in the injured heart

Hadi Khalil,[1] Onur Kanisicak,[1,2] Ronald J. Vagnozzi,[1] Anne Katrine Johansen,[1] Bryan D. Maliken,[1] Vikram Prasad,[1] Justin G. Boyer,[1] Matthew J. Brody,[1] Tobias Schips,[1] Katja K. Kilian,[1] Robert N. Correll,[1,3] Kunito Kawasaki,[4] Kazuhiro Nagata,[4] Jeffery D. Molkentin[1,5]

[1]Department of Pediatrics, Cincinnati Children's Hospital Medical Center, University of Cincinnati, Cincinnati, Ohio, USA
[2]Department of Pathology, University of Cincinnati, Cincinnati, Ohio, USA
[3]Department of Biological Sciences, University of Alabama, Tuscaloosa, Alabama, USA
[4]Institute for Protein Dynamics, Kyoto Sangyo University, Kyoto, Japan
[5]Howard Hughes Medical Institute, Cincinnati Children's Hospital Medical Center, Cincinnati, Ohio, USA

Data Supplement

-Supplemental Figures 1-7

-Supplemental Tables 1-3

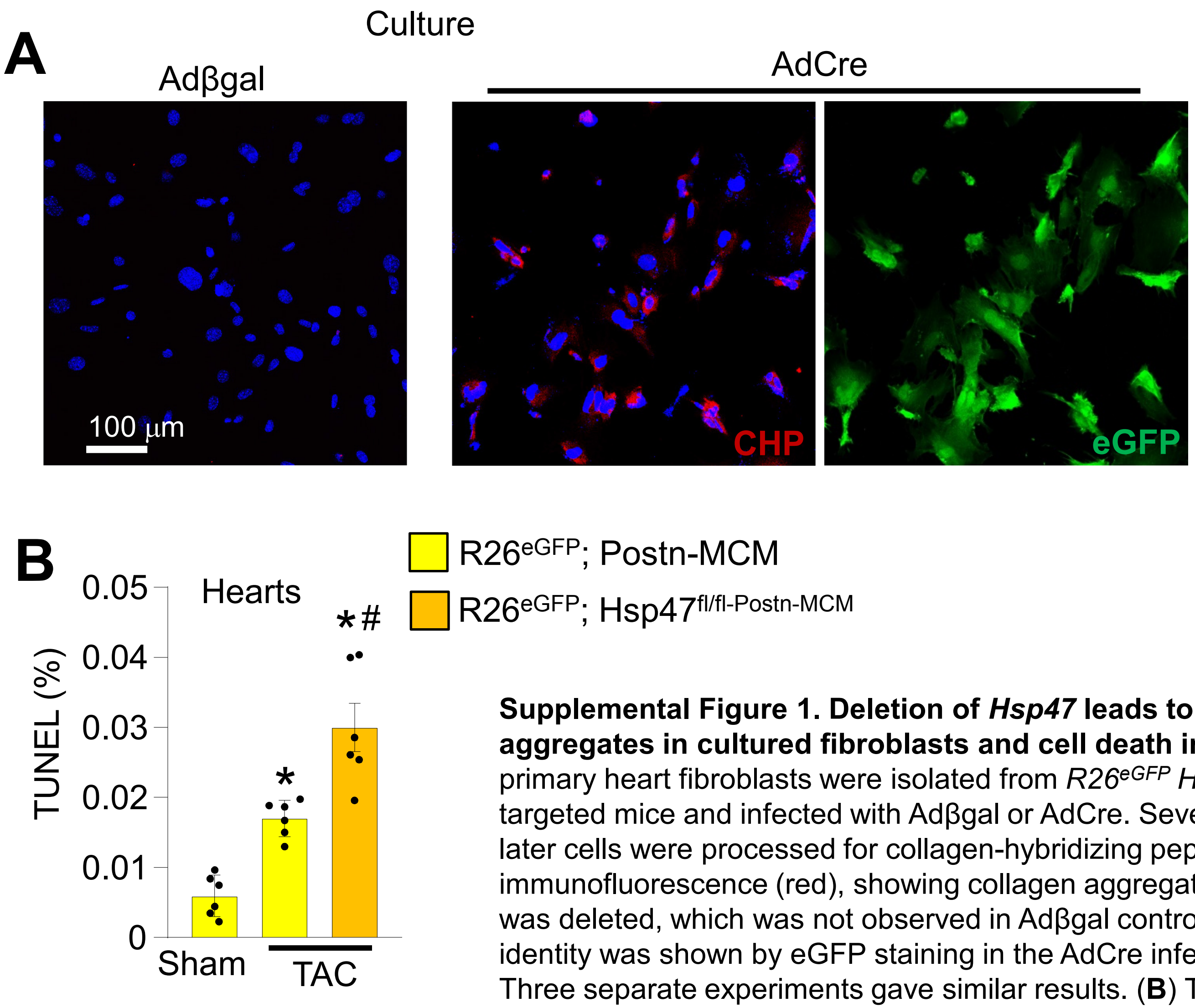

Supplemental Figure 1. Deletion of *Hsp47* leads to collagen aggregates in cultured fibroblasts and cell death in vivo. (**A**) Adult primary heart fibroblasts were isolated from *R26^{eGFP} Hsp47-loxP* targeted mice and infected with Adβgal or AdCre. Seventy-two hours later cells were processed for collagen-hybridizing peptide immunofluorescence (red), showing collagen aggregates when *Hsp47* was deleted, which was not observed in Adβgal controls. Fibroblast identity was shown by eGFP staining in the AdCre infected conditions. Three separate experiments gave similar results. (**B**) TUNEL staining showing cell death levels in hearts of the indicated mice after 4 weeks post TAC. *$P<0.05$ versus Sham; #$P<0.05$ versus *R26^{eGFP} Postn-MCM* control. Number of mice analyzed is shown in the graph.

Supplemental Figure 2

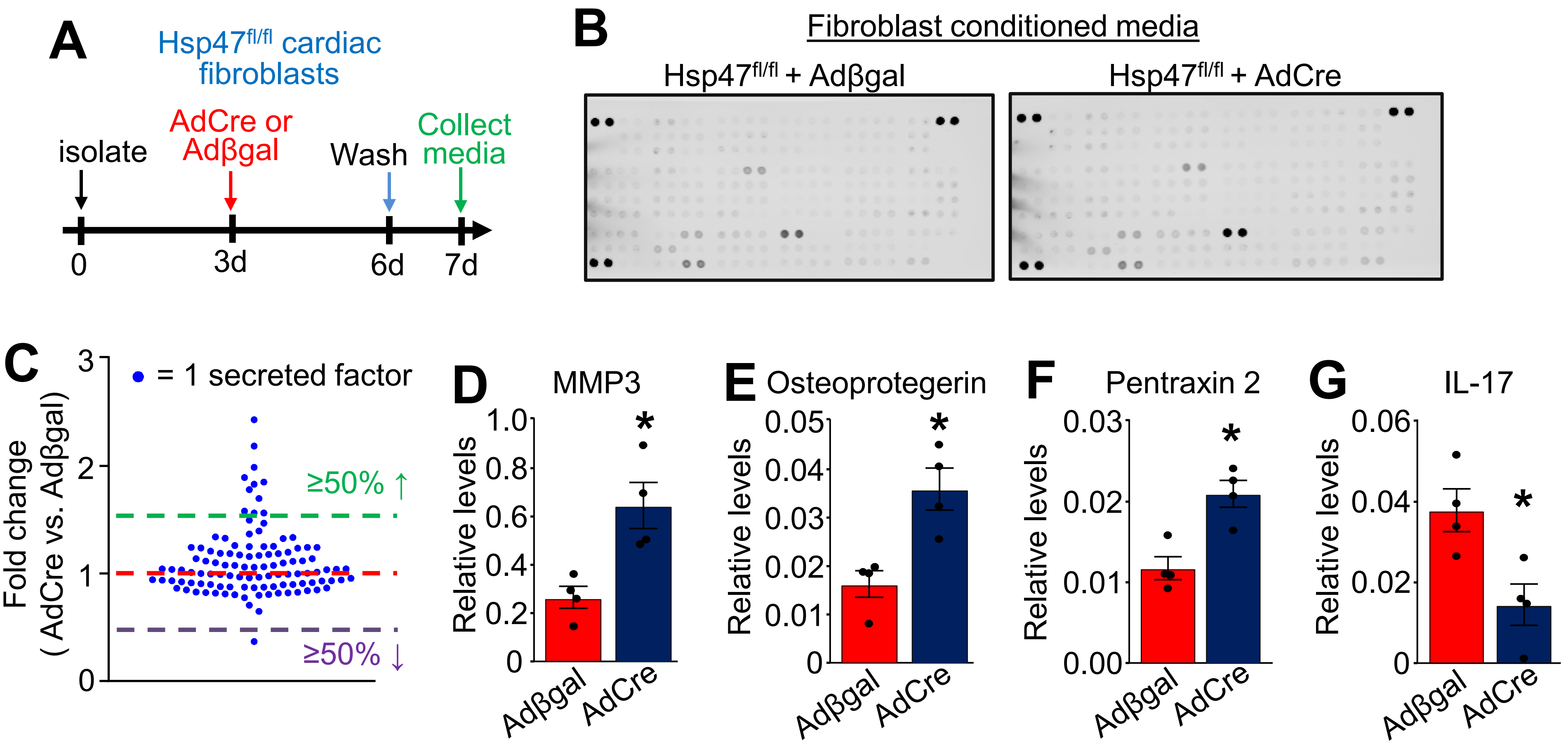

Supplemental Figure 2. Secretome profiling of cardiac fibroblasts deleted for *Hsp47* in vitro. (**A**) Adult primary heart fibroblasts were isolated from *Hsp47-loxP* targeted mice and infected with Adβgal (WT) or AdCre. Seventy-two hours after infection cells were treated with washed and maintained in serum free media for 24 hrs for media collection afterwards. (**B**) Representative membrane scans from sandwich immunoassays showing detection of 111 individual cytokines/secreted factors (in duplicate spots). Membranes were incubated with conditioned media from *Hsp47*$^{fl/fl}$ cardiac fibroblasts infected with either Cre (*Hsp47*-deleted) or β-galactosidase (WT) expressing adenoviruses. (**C**) Quantitation by relative densitometry shows the mean fold change in levels of each secreted protein factor between conditioned media from *Hsp47*-deleted versus WT fibroblasts (*n*=4 per group). (**D-G**) Levels of select secreted factors that showed the largest difference in fold change of protein levels. *p<0.05 by Student's t-test.

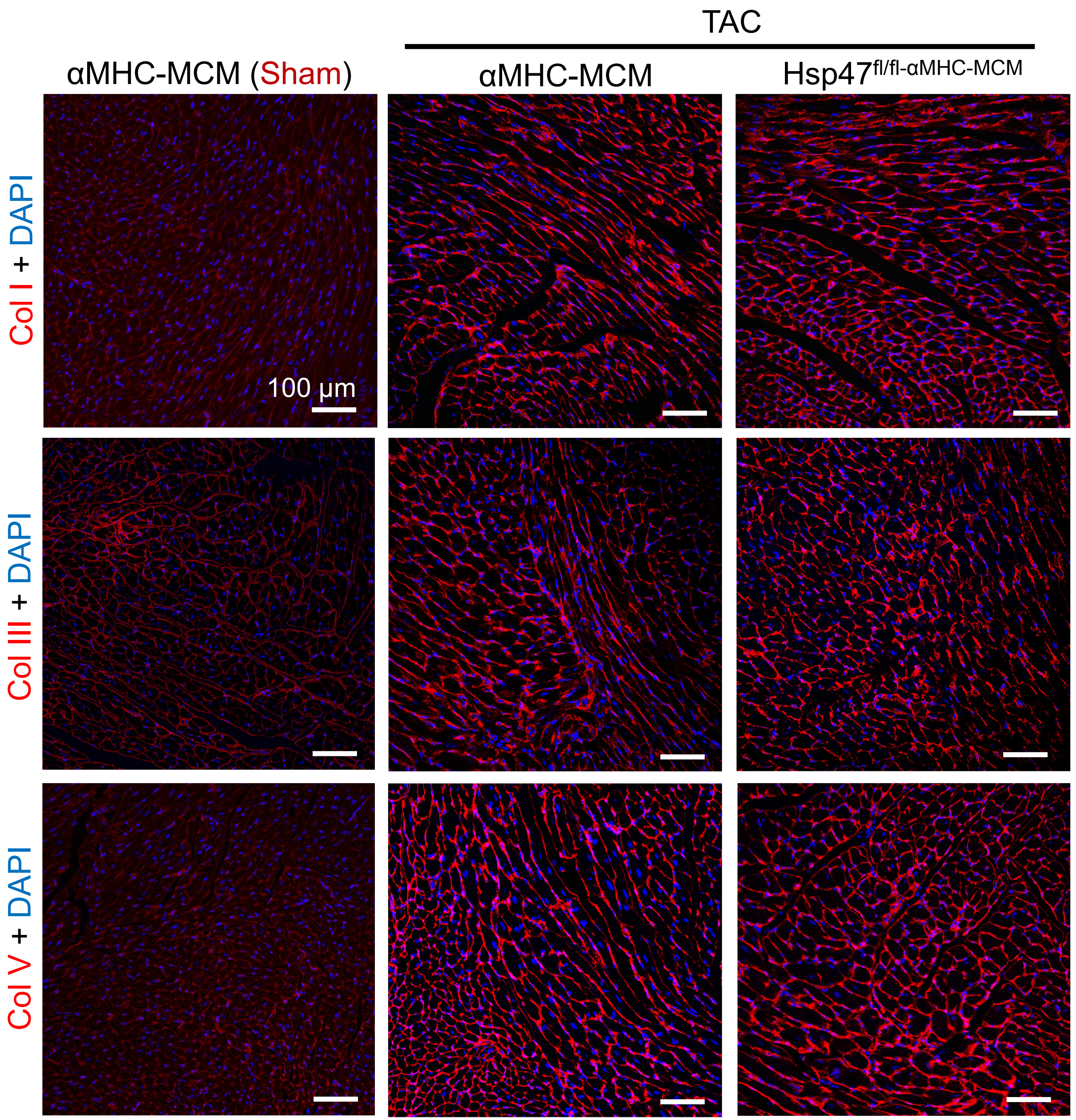

Supplemental Figure 3. Cardiomyocyte-specific deletion of *Hsp47* does not reduce increased collagen deposition after TAC injury. Representative immunohistochemical adult heart images of collagens type-I, -III, and -V from control αMHC-MCM transgenic mice and *Hsp47* cardiomyocyte-specific-deleted mice using this αMHC-MCM transgene. Adult mice were subjected to sham or TAC surgical procedure and then analyzed 4 weeks later. DAPI was used to stain nuclei (blue) while collagens are stained in red fluorescence. Scale bar is 100 µm.

Supplemental Figure 4

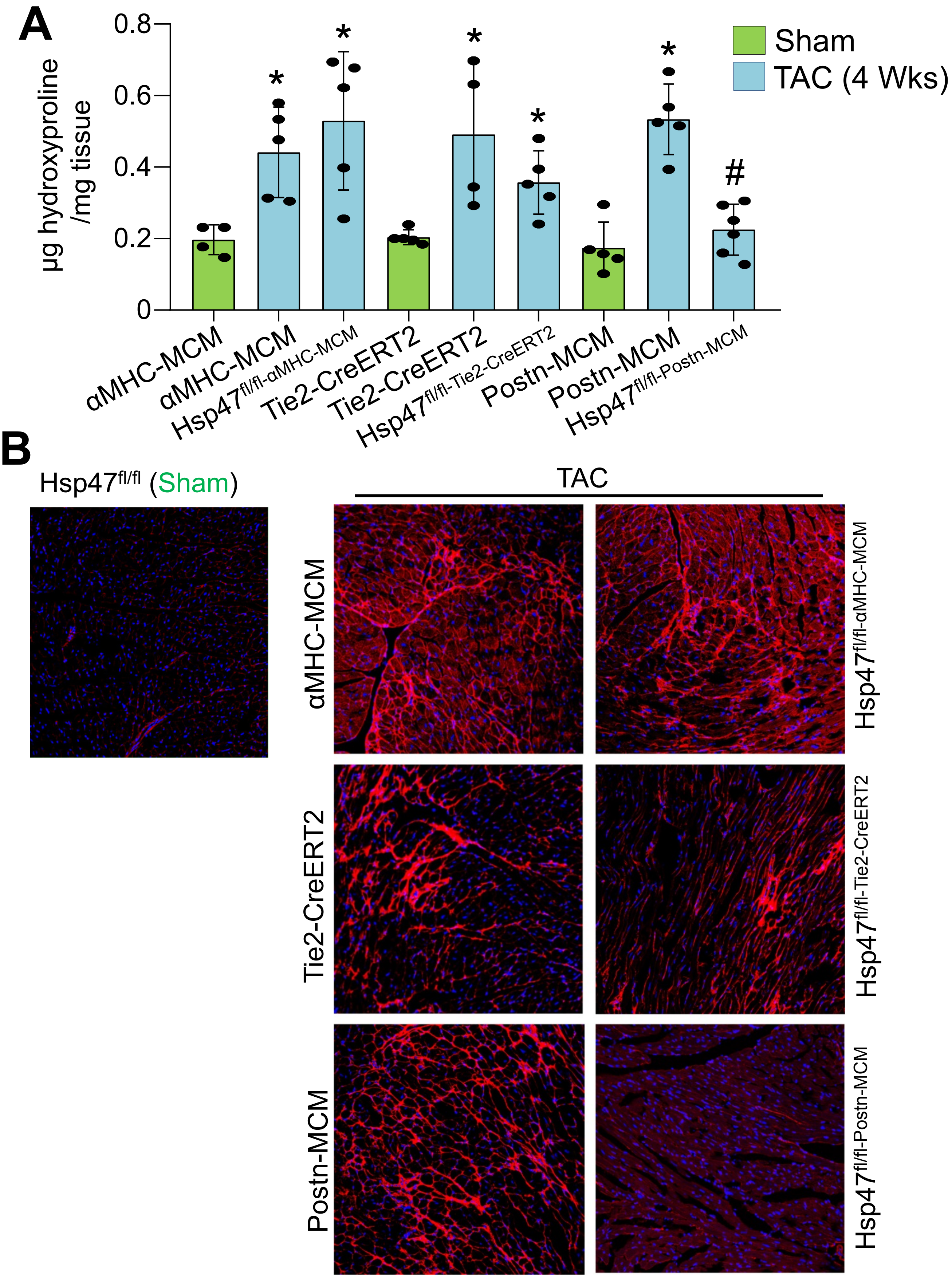

Supplemental figure 4. Fibrosis quantitation in hearts deleted for *Hsp47* in cardiomyocytes, endothelial cells or myofibroblasts. (**A**) Hydroxyproline biochemical assay for fibrosis in whole heart ventricles of the genotypes of mice shown at 4 weeks after TAC injury. n=4-6. *p<0.05 versus any of the 3 sham heart groups; $^{\#}$P<0.05 versus *Postn-MCM* with TAC. (**B**) Unfolded collagens detected from heart histological sections using a biotin-conjugated collagen hybridizing peptide (CHP) that specifically binds unfolded collagen chains in the indicated mouse genotypes of sham and TAC-injured mice over 4 weeks. Scale bar 100 μm. n=5-10 in each group.

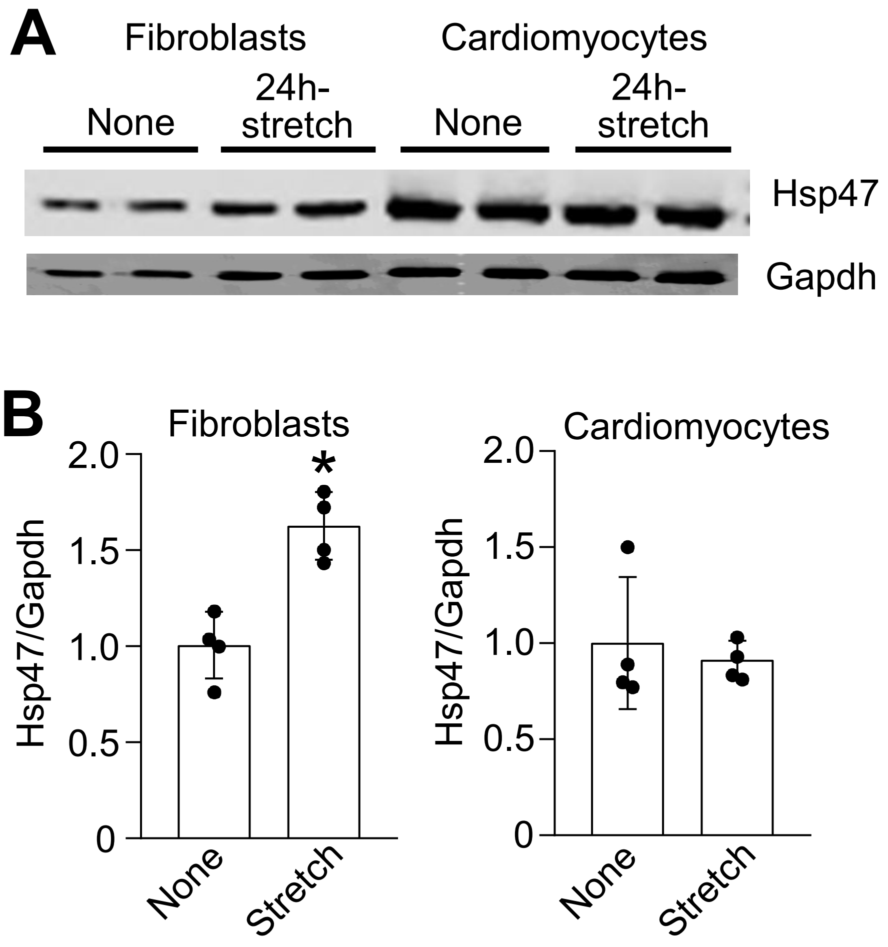

Supplemental Figure 5. Hsp47 is induced in fibroblasts with stretch. Neonatal rat cardiomyocytes and fibroblasts were isolated from the heart and subjected to stretch-induced stimulation. Cells were seeded on 6-well Flexcell culture plates and then a 20% stretch was placed on the silicon membranes. Stretch oscillations were performed continuously for 24 hours at 1.0 Hz. (**A**) Western blot analysis of Hsp47 and Gapdh in these 2 cell-types after 24 hrs stretch or without stretch. Gapdh is shown as a loading control and processing control. (**B**) Quantitation of data in "A" in 6 samples. * $P<0.05$ versus non-stretched cells.

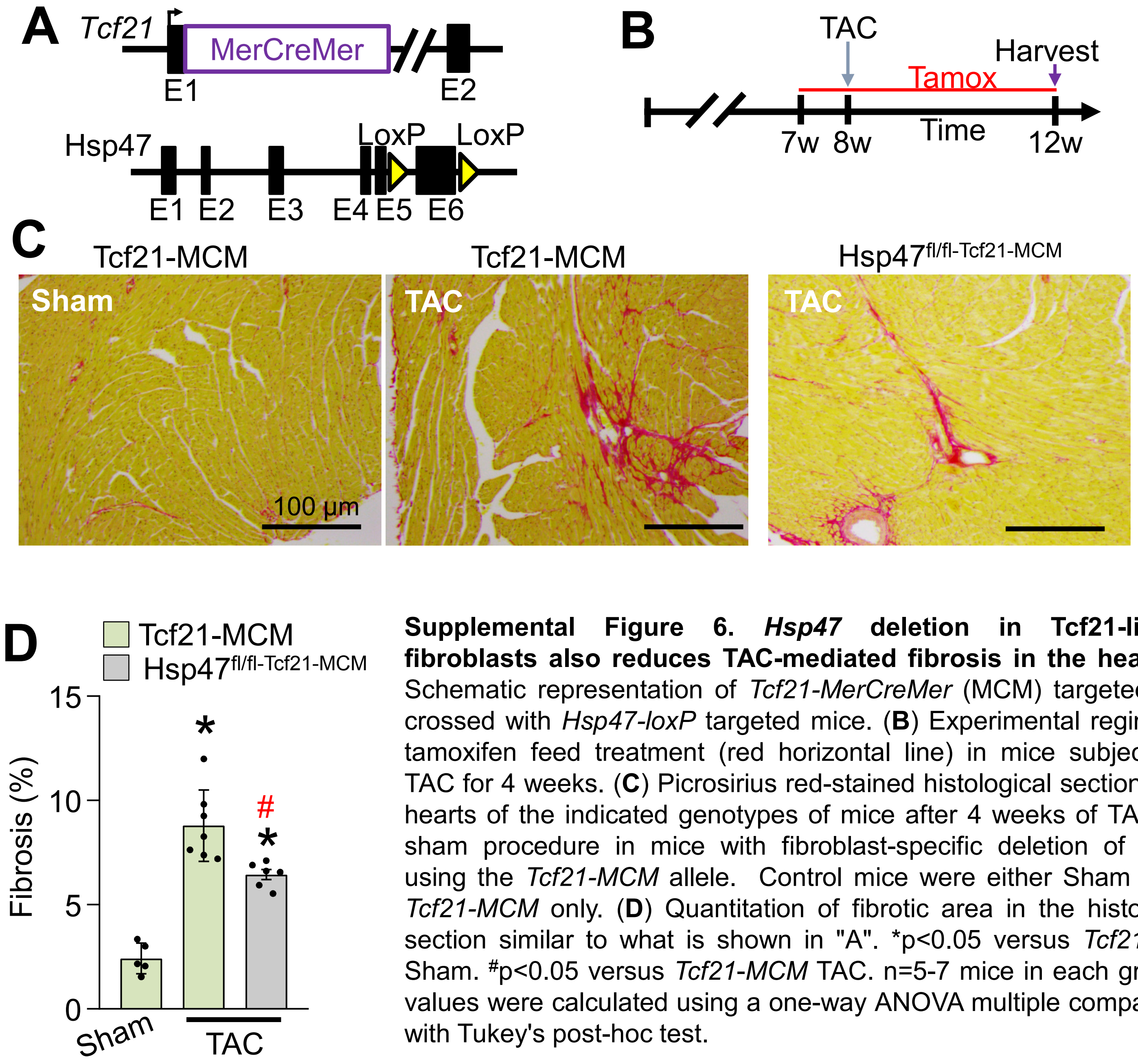

Supplemental Figure 6. *Hsp47* deletion in Tcf21-lineage fibroblasts also reduces TAC-mediated fibrosis in the heart. (**A**) Schematic representation of *Tcf21-MerCreMer* (MCM) targeted mice crossed with *Hsp47-loxP* targeted mice. (**B**) Experimental regimen of tamoxifen feed treatment (red horizontal line) in mice subjected to TAC for 4 weeks. (**C**) Picrosirius red-stained histological sections from hearts of the indicated genotypes of mice after 4 weeks of TAC or a sham procedure in mice with fibroblast-specific deletion of *Hsp47* using the *Tcf21-MCM* allele. Control mice were either Sham and/or *Tcf21-MCM* only. (**D**) Quantitation of fibrotic area in the histological section similar to what is shown in "A". *$p<0.05$ versus *Tcf21*-MCM Sham. #$p<0.05$ versus *Tcf21-MCM* TAC. n=5-7 mice in each group. p values were calculated using a one-way ANOVA multiple comparisons with Tukey's post-hoc test.

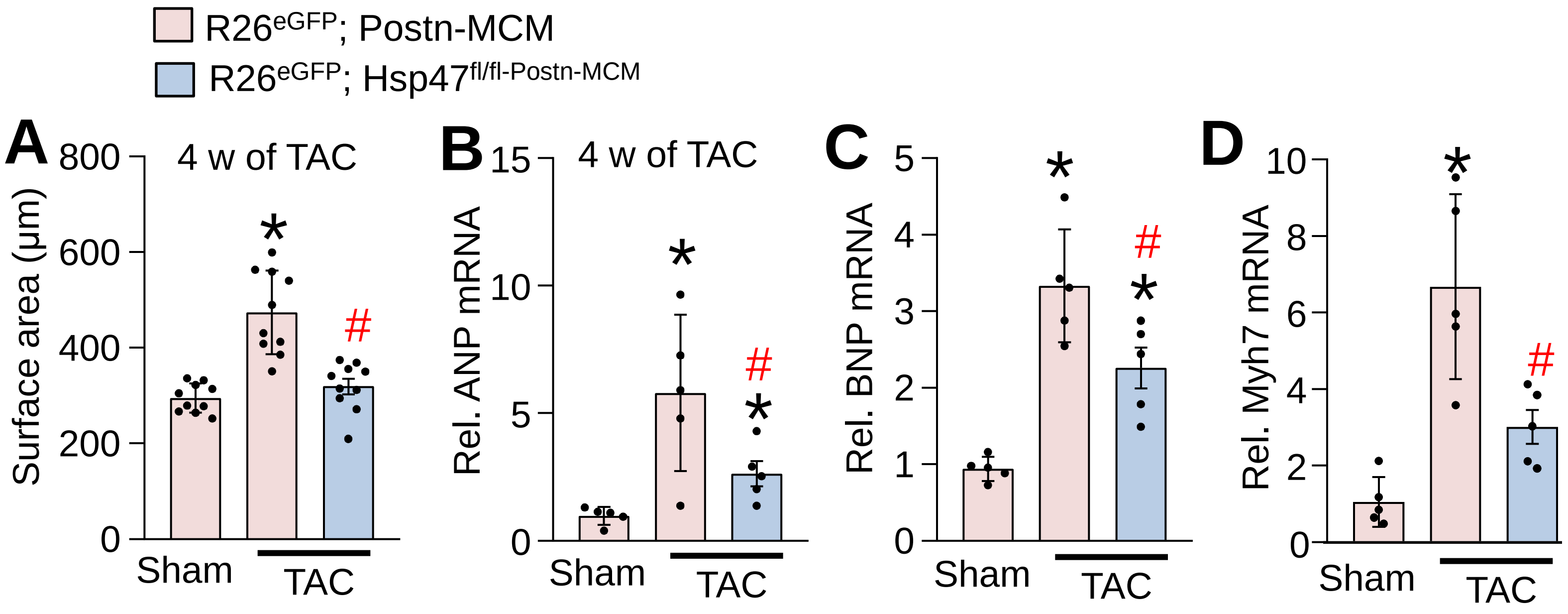

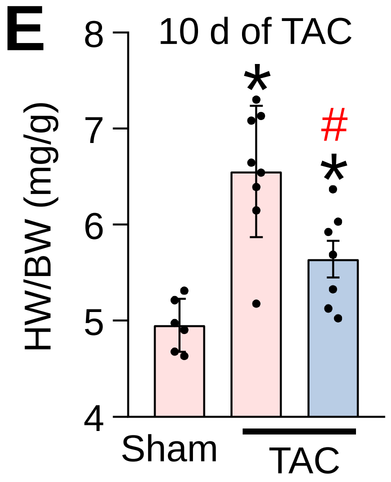

Supplemental Figure 7. Myofibroblast-specific deletion of *Hsp47* reduces hypertrophy after TAC. (**A**) Measurements of cardiomyocyte cell surface area from heart histological sections stained with wheat germ agglutinin-TRITC from the indicated genotypes of mice with 4 weeks of TAC or sham procedure. n=6 (**B,C,D**) Real-time PCR results for mRNA levels of atrial natriuretic peptide (ANP), B-type natriuretic peptide (BNP) and slow twitch myosin heavy chain 7 (Myh7) from the hearts of mice of the indicated genotypes with 4 weeks of TAC or a sham procedure. n=5. (**E**) heart-weight normalized to body-weight in sham or TAC-operated mice after 10 days in the indicated genotypes of mice. *P<0.05 versus *R26^{eGFP} Postn-MCM* sham; #P<0.05 versus *R26^{eGFP} Postn-MCM* TAC-operated. P values one-way ANOVA multiple comparisons with post-hoc Tukey HSD.

Table 1. Representative gene groups from RNAseq upregulated in Hsp47 null cardiac fibroblasts vs WT with 4 weeks for TAC

	Symbol	Accession ID	Gene title	Fold	p-value
Chondrocyte like	Cthrc1	MGI:1915838	collagen triple helix repeat containing 1	4.05	0.0001
	Cilp	MGI:2444507	cartilage intermediate layer protein, nucleotide pyrophosphohydrolase	3.43	0.0003
	Colec12	MGI:2152907	collectin sub-family member 12	1.55	0.0001
	Comp	MGI:88469	cartilage oligomeric matrix protein	12.97	0.0035
	Chad	MGI:1096866	chondroadherin	5.38	0.0020
	Chadl	MGI:3036284	chondroadherin-like	1.80	0.0396
Collagen assembly	Aspn	MGI:1913945	asporin	1.97	0.0027
	Sdc4	MGI:1349164	syndecan 4	1.90	0.0103
	Tnmd	MGI:1929885	tenomodulin	4.23	0.0018
	Fmod	MGI:1328364	fibromodulin	5.33	0.0009
	Fbln5	MGI:1346091	fibulin 5	1.95	0.0017
	Smtn	MGI:1354727	smoothelin	2.07	0.0030
	Emilin1	MGI:1926189	elastin microfibril interfacer 1	4.24	0.0020
ECM	Postn	MGI:1926321	periostin, osteoblast specific factor	21.32	3.25E-06
	Col8a2	MGI:88464	collagen, type VIII, alpha 2	4.22	0.0035
	Col12a1	MGI:88448	collagen, type XII, alpha 1	3.03	0.0025
	Col22a1	MGI:1916950	collagen, type XXII, alpha 1	5.37	6.88E-05
	Fndc1	MGI:1915905	fibronectin type III domain containing 1	2.26	0.0535
	Fndc4	MGI:1917195	fibronectin type III domain containing 4	2.21	0.0100
ECM Modulation	Mmp2	MGI:97009	matrix metallopeptidase 2	2.10	0.0010
	Mmp3	MGI:97010	matrix metallopeptidase 3	2.35	0.1490
	Mmp19	MGI:1927899	matrix metallopeptidase 19	1.48	0.1005
	Mmp23	MGI:1347361	matrix metallopeptidase 23	2.61	0.1377
	Adam9	MGI:105376	a disintegrin and metallopeptidase domain 9 (meltrin gamma)	1.67	0.0117
	Adamts6	MGI:1347348	a disintegrin-like and metallopeptidase (reprolysin type) with thrombospondin type 1 motif	2.07	0.0072
	Adamtsl2	MGI:1925044	ADAMTS-like 2	3.33	0.0538
	Serpina3n	MGI:105045	serine (or cysteine) peptidase inhibitor, clade A, member 3N	1.87	0.0007
	Serpinf1	MGI:108080	serine (or cysteine) peptidase inhibitor, clade F, member 1	2.24	0.0141

	Lox	MGI:96817	lysyl oxidase	2.18	0.1359
	Loxl1	MGI:106096	lysyl oxidase-like 1	3.26	0.0883
Cell Adhesion	Itgb7	MGI:96616	integrin beta 7 [Source:MGI Symbol;Acc:]	1.72	0.0547
	Itga11	MGI:2442114	integrin alpha 11 [Source:MGI Symbol;Acc:]	3.32	0.0004
	Ctgf	MGI:95537	connective tissue growth factor [Source:MGI Symbol;Acc:]	2.63	0.0003
	Cdh2	MGI:88355	cadherin 2 [Source:MGI Symbol;Acc:]	2.01	0.0036
	Chl1	MGI:1098266	cell adhesion molecule with homology to L1CAM	-1.84	0.0098
	Cdh7	MGI:2442792	cadherin 7, type 2	-2.21	0.0028
	Cadm1	MGI:1889272	cell adhesion molecule 1	-1.69	0.0415
	Adam12	MGI:105378	a disintegrin and metallopeptidase domain 12 (meltrin alpha)	-1.50	0.0250
	Canx	MGI:88261	calnexin [Source:MGI Symbol;Acc:]	3.87	0.0051
Secreted factors	Ang	MGI:88022]	angiogenin, ribonuclease, RNase A family, 5	3.50	1.94E-05
	Angptl7	MGI:3605801	angiopoietin-like 7	4.29	0.0004
	Ptn	MGI:97804	pleiotrophin	2.23	0.0081
	Sfrp2	:MGI:108078	secreted frizzled-related protein 2	3.97	0.0018
	Ccl3	MGI:98260]	chemokine (C-C motif) ligand 3	2.50	0.0537
	Fbrs	MGI:104648	fibrosin	2.29	0.0325
	Cx3cl1	MGI:1097153	chemokine (C-X3-C motif) ligand 1	4.82	0.0008
	Clec4n	MGI:1861231]	C-type lectin domain family 4, member n	3.42	0.0009
TGFB signaling	Tgfb1	MGI:98725	transforming growth factor, beta 1	2.59	0.0113
	Ltbp2	MGI:99502	latent transforming growth factor beta binding protein 2	2.84	0.0030
	Ltbp3	MGI:1101355	latent transforming growth factor beta binding protein 3	2.54	0.0120
	Nlgn2	MGI:2681835	neuroligin 2	2.78	0.0028
	Furin	MGI:97513	furin (paired basic amino acid cleaving enzyme)	1.94	0.0028
	Bmp6	MGI:88182	bone morphogenetic protein 6	2.25	0.0025

Table 2. List of primers used for qPCR to quantify the designated mRNA species

Gene ID	Sense (5'-3')	Antisense (5'-3')
Col1a1	AATGGCACGGCTGTGTGCGA	AACGGGTCCCCTTGGGCCTT
Col1a2	GCAGGTTCACCTACTCTGTCCT	CTTGCCCCATTCATTTGTCT
Col3a1	TCCCCTGGAATCTGTGAATC	TGAGTCGAATTGGGGAGAAT
Col4a1	TTAAAGGACTCCAGGGACCAC	CCCACTGAGCCTGTCACAC
Col5a1	GGGCAGAAGGGAAGCAAG	GGCCTTGAGGACCGGTAG
Col6a1	GCAAGGATGAGCTGGTCAA	GTCCACGTGCTCTTGCATC
Col11a1	AAAACGAAACGGTGGATCTG	TGTTTGTGCAAAATCCTGTTG
Vim	GCCGAAAGCACCCTGCAGTCA	GCCTGCAGCTCCTGGATCTCTTCA
Acta2	TGACGCTGAAGTATCCGATAGA	CGAAGCTCGTTATAGAAAGAGTGG
Tnc	TCCCCGGGACTGTAGCCAGC	CGTGGCAGTCACTGGGGCAG
Fbn	TGACTGGCCCACACGTGCATAG	TGACATTGACCCCTTGTTGACAGGA
Fn	AAGGCTGGATGATGGTGGAC	TGAAGCAGGTTTCCTCGGTTG
Timp1	GCAACTCGGACCTGGTCATAA	CGGCCCGTGATGAGAAACT
Mmp9	AATCTCTTCTAGAGACTGGGAAGGAG	AGCTGATTGACTAAAGTAGCTGGA
Mmp23	CGACTTCTGCTACGAATTCCC-3	TTGGCGATGATGCTCAGGTG-3
Zeb1	GCTGGCAAGACAACGTGAAAG	GCCTCAGGATAAATGACGGC
Chd1	AGCTCTAAGGACAGTGGTCAT	CAGTGCTTTACATTCCTCGGT
Snai1	GACCACTATGCCGCGCTCTT	TCGCTGTAGTTAGGCTTCCGATT
cdkn1a	CCTGGTGATGTCCGACCTG	CCATGAGCGCATCGCAATC
Chad	CTGGGTCTTTATGCTGTTGCT	CCGTCTTGAGTTGTCGCAGA
Cthrc1	CAGTTGTCCGCACCGATCA	GGTCCTTGTAGACACATTCCATT
Aspn	AAGGAGTATGTGATGCTACTGCT	ACATTGGCACCCAAATGGACA
Tnmd	ACACTTCTGGCCCGAGGTAT	GACTTCCAATGTTTCATCAGTGC
Fmod	AGCAGTCCACCTACTACGACC	CAGTCGCATTCTTGGGGACA
Sdc4	TTTGCCGTTTTCCTGATCCTG	TTGCCCAAGTCGTAACTGCC
Postn	ACGGAGCTCAGGGCTGAAGATG	GTTTGGGCCCTGATCCCGAC
Mmp3	ACCAACCTATTCCTGGTTGCTGCT	ATGGAAACGGGACAAGTCTGTGGA
Lox	TGATGCCAACACCCAGAGGA	CGAATGTCACAGCGCACAAC
Itga11	TGCCCCAATGGAAACCAATG	CACTCGTGCGACCAGAGAG
Ptn	CTCTGCACAATGCTGACTGTC	ACAGCTTCTTACCTTGAGGCTT
Tgfb2	TCGACATGGATCAGTTTATGCG	CCCTGGTACTGTTGTAGATGGA
Furin	TGGTTGCTATGGGTGGTCG	CCAGAAGTGGTAATAGTCACCGA
Gapdh	TTGTCTCCTGCGACTTCAAC	GTCATACCAGGAAATGAGCTTG
Cdh7	GGGCAGGAGGGCTAGGCATTAACCT	TAGCTGTCCTCCTGGAACAGGGCGT
Adam12	AGACGTGCTGACTGTGCAAC	CCGTGTGATTTCGAGTGAGAGA
Myh7	ACAACCCCTACGATTATGCGT	ACGTCAAAGGCACTATCCGTG
ANP	GCTTCCAGGCCATATTGGAG	GGGGGCATGACCTCATCTT
BNP	GAGGTCACTCCTATCCTCTGG	GCCATTTCCTCCGACTTTTCTC

Table 3. Echocardiographic assessment of the heart 4 weeks after TAC (supports Fig 1, C-M and Fig 2)

Genotype	Injury	IVSd (cm)	IVSs (cm)	LVIDd (cm)	LVIDs (cm)	LVPWd (cm)	LVPWs (cm)	FS (%)
**βMHC-Cre**	Sham	0.074±0.003	0.105±0.003	0.383±0.008	0.247±0.007	0.085±0.005	0.108±0.008	35.38±0.39
**Hsp47$^{fl/fl-bMHC-Cre}$**	Sham	0.072±0.002	0.107±0.004	0.390±0.008	0.257±0.005	0.083±0.006	0.117±0.006	37.16±0.10
**βMHC-Cre**	TAC	0.081±0.001	0.123±0.005	0.446±0.015	0.316±0.012	0.109±0.004	0.138±0.006	29.14±1.19
**Hsp47$^{fl/fl-bMHC-Cre}$**	TAC	0.083±0.001	0.124±0.003	0.442±0.018	0.309±0.014	0.105±0.007	0.144±0.005	29.44±0.51
**αMHC-MCM**	Sham	0.072±0.004	0.101±0.006	0.387±0.0229	0.265±0.019	0.087±0.005	0.106±0.005	36.54±0.55
**αMHC-MCM**	TAC	0.079±0.005	0.129±0.006	0.434±0.0180	0.306±0.013	0.144±0.005	0.126±0.010	26.10±0.65
**Hsp47$^{fl/fl-\alpha MHC-MCM}$**	TAC	0.084±0.005	0.127±0.008	0.429±0.0159	0.308±0.008	0.110±0.004	0.131±0.004	27.18±0.57
**Tie2-CreERT2**	Sham	0.072±0.008	0.102±0.008	0.392±0.012	0.253±0.010	0.079±0.004	0.104±0.004	38.74±0.79
**Tie2-CreERT2**	TAC	0.074±0.003	0.126±0.006	0.308±0.020	0.303±0.013	0.104±0.003	0.133±0.007	28.18±0.88
**Hsp47$^{fl/fl-Tie2-CreERT2}$**	TAC	0.079±0.001	0.131±0.003	0.442±0.018	0.309±0.014	0.099±0.007	0.138±0.005	29.44±0.51
